# Building Feedback-Regulation System Through Atomic Design for Highly Active SO_2_ Sensing

**DOI:** 10.1007/s40820-024-01350-3

**Published:** 2024-02-27

**Authors:** Xin Jia, Panzhe Qiao, Xiaowu Wang, Muyu Yan, Yang Chen, Bao-Li An, Pengfei Hu, Bo Lu, Jing Xu, Zhenggang Xue, Jiaqiang Xu

**Affiliations:** 1 NEST Lab, Department of Chemistry, College of Sciences, Shanghai University, Shanghai, 200444 People’s Republic of China; 2 Shanghai Synchrotron Radiation Facility, Zhangjiang Lab, Shanghai Advanced Research Institute, Chinese Academy of Sciences, Shanghai, 201204 People’s Republic of China; 3 School of Chemistry and Materials Science, University of Science and Technology of China, Hefei, 230026 People’s Republic of China; 4 Key Laboratory of Organic Compound Pollution Control Engineering (MOE), School of Environmental and Chemical Engineering, Shanghai University, Shanghai, 200444 People’s Republic of China; 5 Shanghai University, Instrumental Analysis & Research Center of Shanghai University, Shanghai, 200444 People’s Republic of China

**Keywords:** Feedback-regulation system, Atomic interface, SO_2_ sensor, Single-atom sensing mechanism, Intelligent-sensing array

## Abstract

Reasonably constructing an atomic interface is pronouncedly essential for surface-related gas-sensing reaction. Herein, we present an ingenious feedback-regulation system by changing the interactional mode between single Pt atoms and adjacent S species for high-efficiency SO_2_ sensing. We found that the single Pt sites on the MoS_2_ surface can induce easier volatilization of adjacent S species to activate the whole inert S plane. Reversely, the activated S species can provide a feedback role in tailoring the antibonding-orbital electronic occupancy state of Pt atoms, thus creating a combined system involving S vacancy-assisted single Pt sites (Pt-Vs) to synergistically improve the adsorption ability of SO_2_ gas molecules. Furthermore, in situ Raman, ex situ X-ray photoelectron spectroscopy testing and density functional theory analysis demonstrate the intact feedback-regulation system can expand the electron transfer path from single Pt sites to whole Pt-MoS_2_ supports in SO_2_ gas atmosphere. Equipped with wireless-sensing modules, the final Pt_1_-MoS_2_-def sensors array can further realize real-time monitoring of SO_2_ levels and cloud-data storage for plant growth. Such a fundamental understanding of the intrinsic link between atomic interface and sensing mechanism is thus expected to broaden the rational design of highly effective gas sensors.

**Supplementary Information:**

The online version contains supplementary material available at 10.1007/s40820-024-01350-3.

## Highlights

- Feedback-regulation system is established between single Pt sites and MoS_2_ supports
- The Pt_1_-MoS_2_-def can expand the electron transfer path from single Pt sites to whole Pt-MoS_2_ supports in SO_2_ gas atmosphere.
- The Pt_1_-MoS_2_-def sensors exhibit high SO_2_ responses and extremely low limit of detection (3.14% to 500 ppb SO_2_) at room temperature.
- The Pt_1_-MoS_2_-def sensors array can realize real-time monitoring of SO_2_ for plant growth.

**Supplementary Information:**

The online version contains supplementary material available at 10.1007/s40820-024-01350-3.

## Graphical abstract

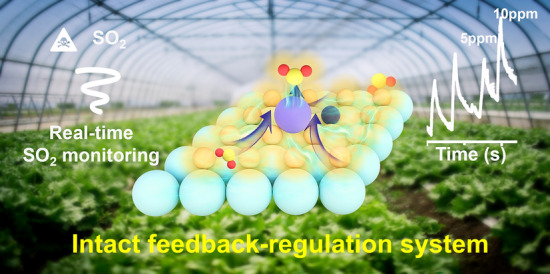

## Introduction

Sulfur dioxide (SO_2_) constitutes a significant proportion of harmful air pollutants, and the main sources of SO_2_ gas include the combustion of sulfur-containing fuels in the manufacturing and construction sectors and volcanic eruptions and forest fires [1, 2]. Additionally, sulfur dioxide not only contributes to acid rain but also has detrimental effects on both human and plant growth [3]. Research has found that the sensitivity to SO_2_ gas varies significantly among different plants, sensitive plants exposed to an atmosphere with 0.5 ppm of SO_2_ exhibit chloroplast damage and photosynthesis stagnation after 8 h. When exposed to an atmosphere with 1–4 ppm of SO_2_, damage occurs within 3 h [4].

Sulfur dioxide gas sensors are wildly applied in many fields, such as comprehensive treatment of environmental pollution [5], monitoring the quality of the healthy environment inspection [6, 7] and improving plant productivity [8]. Conventionally, SO_2_ concentrations are measured using two optical tracking technologies, infrared radiation spectroscopy or UV absorbance spectroscopy. In the quest to achieve continuous monitoring of harmful pollution, solid-state resistive gas sensors employing semiconducting have been the widest spread in gas-sensing application owing to their compactness and versatility. In particular, the room temperature SO_2_ sensors based on two-dimensional materials have gradually evolved into the development trend on account of their low power consumption (compared to required high working temperature for most oxide–semiconductor sensors) and small sizes (no integrated limitation for microheater and sensing layer) [9–11]. Given lacking supplied energy at high operation temperature to activate the sensing-layer, the performances of room-temperature SO_2_ sensors are more dependent on the intrinsic activity of sensing materials. Compared with mental oxides semiconductor (MOS) sensors that work at high temperatures, molybdenum disulfide has been extensively studied for its excellent gas-sensing property at the room temperature. For example, previous study has demonstrated that doping transition metal to inner MoS_2_ supports can markedly improve the SO_2_ sensing property [12]. Therefore, engineering highly active sensitive materials to meet the requirements of high sensitivity and low limit of detection (LOD) for room-temperature SO_2_ detection is crucial, yet challenging.

In recent years, single-atom catalysts (SACs) have been considered as potential candidates for high-performance gas sensors due to their maximum utilization efficiency of metal atoms and uniform unsaturated configuration of active centers [13–17]. Generally, the single metal centers (especially for Pd, Pt, Au and so on) are considered as highly active adsorption and reactive sites during the gas-sensing process [18–22]. The sensitization action mainly involves chemical and electronic effects. On the one hand, the unsaturated metal center can reinforce the bonding process to objective gas molecules and lead potential activation of gas molecules [23, 24]. On the other hand, the metal center can also induce more electron transfer with the target gas molecules to improve the sensing response [25]. However, this sensitization action may result in a major electron concentration localized at these single-metal sites [13, 16, 26, 27], while the participation of support materials is often ignored. Considering the extremely low single metal loadings, activating the main support materials are thus considered as a crucial strategy to further enhance the gas-sensing performance. Meanwhile, the interactive status between single-atom centers and the whole activated supports may trigger the linkage effect to synergistically enhance the gas adsorption and electronic transform process. This route of reaction mechanism is thus apparently different from that of traditional SACs. However, engineering active support-associated SACs and deeply exploring the gas-sensing action are still rarely studies.

**Fig. 1:**
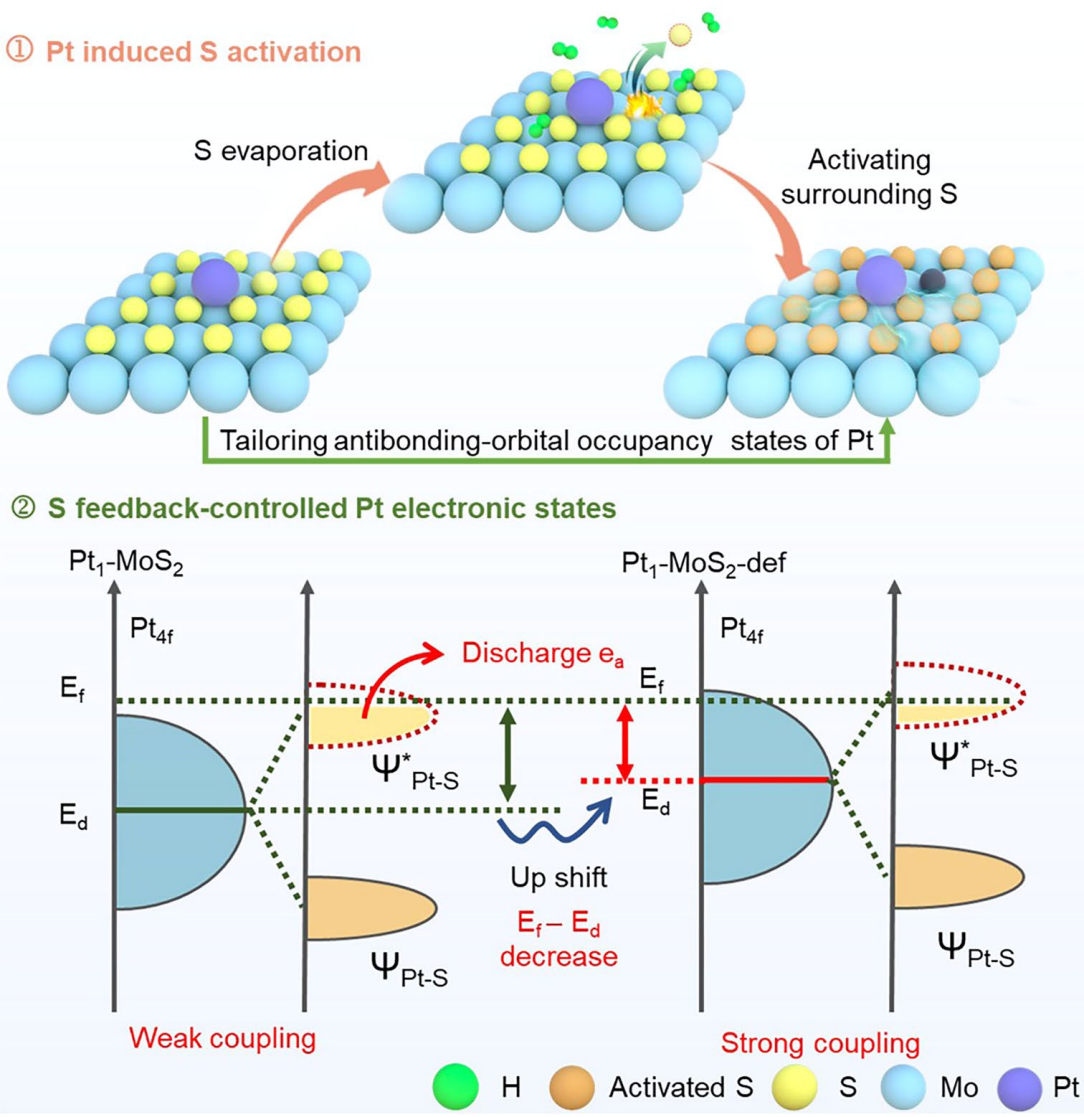
The schematic of the feedback-regulation system in the Pt_1_-MoS_2_-def

**Fig. 2:**
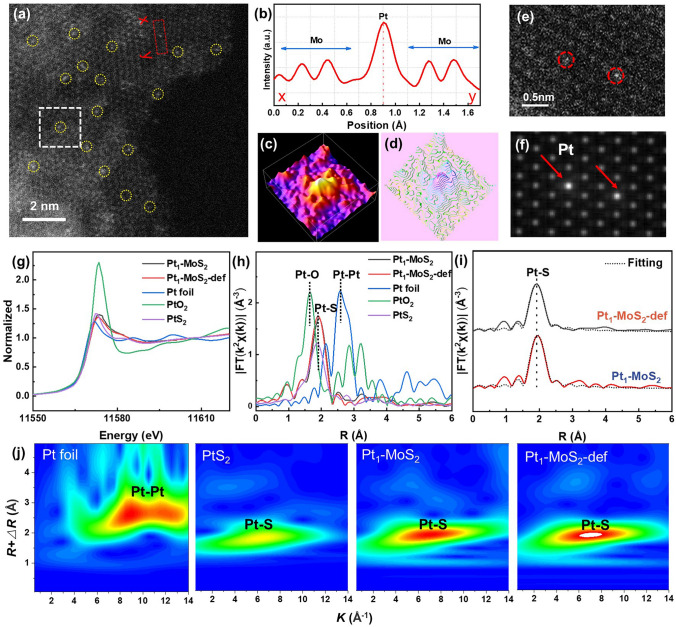
Characterization of Pt_1_-MoS_2_ and Pt_1_-MoS_2_-def. **a** HAADF-STEM image of Pt_1_-MoS_2_-def. **b** The intensity profiles obtained in regions X–Y in (**a**). **c, d** The atom-overlapping Gaussian-function fitting images of selective area scan (measured from white frame of (**a**). **e, f** HAADF-STEM image of Pt_1_-MoS_2_-def and its simulated image. **g** Pt L-edge XANES spectra. **h** FT-EXAFS spectra and **i** corresponding fitting curve of Pt L_3_ edge. **j** Wavelet transform patterns of Pt foil, PtS_2_, Pt_1_-MoS_2_ and Pt_1_-MoS_2_-def

Here, we present an ingenious feedback-regulation system by changing the interactional mode between single Pt sites and MoS_2_ supports for high-efficiency room-temperature SO_2_ sensing. Firstly, the introduced single Pt atoms can activate the whole S species on supports to form S vacancy-assisted single Pt sites (Pt-Vs) at reductive atmosphere (Fig. 1-①). Reversely, the activated S species can provide a feedback role in tailoring the antibonding-orbital electronic occupancy state of Pt atoms (Fig. 1-②). Detailedly, the assistant sulfur vacancy can elevate the d-band center position of the single Pt sites and reduce the occupation state of the Pt–S antibonding orbital, thus increasing the strength of the Pt–S bond and further improving the SO_2_ adsorption. Gas adsorption/desorption experiments and in situ gas adsorption breakthrough experiments demonstrate sulfur vacancy-aided Pt_1_-MoS_2_ catalysts (Pt_1_-MoS_2_-def) exhibit the strongest SO_2_ gas adsorption capacity. Ex situ X-ray photoelectron spectroscopy (XPS) and in situ Raman spectroscopy further experimentally confirm that the intact feedback-regulation system can expand the electron transfer path from single Pt sites to whole Pt_1_-MoS_2_ supports in SO_2_ gas atmosphere. This thus endows Pt_1_-MoS_2_-def sensors extremely low limit of detection (500 ppb) and high response sensitivity (3.14% to 500 ppb SO_2_) at room temperature. At the same time, equipped with Bluetooth intelligent monitoring modules, the Pt_1_-MoS_2_-def sensors array can further realize real-time monitoring of SO_2_ levels and cloud-data transmission/storage for plant growth.

## Experimental Section

### Materials

Chloroplatinic acid (H_2_PtCl_6_), ascorbic acid (AA), ammonium molybdate tetrahydrate ((NH_4_)_6_Mo_7_O_24_·4H_2_O), aniline, l-Cysteine, hydrochloric acid (HCl) and ethanol (C_2_H_5_OH) were analytical grade and purchased from Shanghai Chemical Corp. All reagents in the experiment were used without any further purification. All solutions were prepared in deionized water. Gases including hydrogen sulfide (H_2_S), nitrous oxide (N_2_O), xylene (C_8_H_10_), nitrogen dioxide (NO_2_), nitric oxide (NO), hydrogen (H_2_), ammonia (NH_3_) and sulfur dioxide (SO_2_) were obtained from Shanghai Shenkai Gases Technology Co., Ltd.

### Preparation of Trimolybdate, MoS_2_, MoS_2_-def, Pt_1_-MoS_2_, Pt_1_-MoS_2_-def and Pt NPs-MoS_2_

#### ***Preparation of Aniline Trimolybdate (MoO***_***3***_***-ANE)***

The MoO_3_-ANE was synthesized following a previous report [28] with minor modification. First, 2.48 g of (NH_4_)_6_Mo_7_O_24_·4H_2_O was dissolved in 50 mL of deionized water and 3.27 g of aniline was added. Then, 1 M HCl aqueous solution was added dropwise with magnetic stirring at room temperature until a white precipitate appeared (pH 4–5). After a reaction at 50 °C for 2 h, the product was filtered and thoroughly washed with ethanol, and then dried at 70 °C.

#### ***Preparation of Flower-Shaped MoS***_***2***_

The obtained MoO_3_-ANE (0.1 g) was dispersed in a homogenous solution containing deionized water (15 mL) and l-Cysteine (0.2836 g). The dispersion solution was subsequently transferred to 50 mL Teflon-lined stainless-steel autoclaves and maintained at 200 °C for 14 h. The obtained samples were rinsed with ethanol and water and oven-dried at 70 °C for one day. Then, as-prepared powder was calcined in a tube furnace at 400 °C for 1 h at the heating rate of 2 °C min^−1^ under N_2_ atmosphere.

#### ***Synthesis of MoS***_***2***_***-def***

The obtained black powder of MoS_2_ (200 mg) was treated in a tube furnace at 100 °C for 1 h under the 5% H_2_/Ar_2_ atmosphere at the heating rate of 2 °C min^−1^. The final product MoS_2_-def was collected.

#### ***Synthesis of Pt***_***1***_***-MoS***_***2***_***, Pt***_***1***_***-MoS***_***2***_***-def and Pt NPs-MoS***_***2***_

The obtained black powder of MoS_2_ (200 mg) was dispersed in aqueous solution of deionized water (25 mL) and ethanol (25 mL). An aqueous solution of chloroplatinic acid (10 mg mL^−1^, 0.2 mL) was subsequently added into the dispersion and stirred for 24 h at room temperature. The precipitate was separated, washed and dried under vacuum at 70 °C for 24 h. The as-above precipitate was treated in a tube furnace at 100 °C for 1 h under the 5% H_2_/Ar atmosphere at the heating rate of 2 °C min^−1^. The final product Pt_1_-MoS_2_-def was collected. Pt_1_-MoS_2_ was prepared with the same synthesis procedure of Pt_1_-MoS_2_-def except the tube furnace was not treated. The decoration of Pt (0.1 wt%) NPs was achieved through in situ reduction method. 200 mg as-prepared MoS_2_ powder were subsequently dispersed in 25 mL ethanol and 25 mL deionized water. An aqueous solution of chloroplatinic acid (10 mg mL^−1^, 30 μL) and 1 mL AA solution (0.1 M) were subsequently added into the dispersion and stirred for 24 h at room temperature. The product was precipitated by centrifugation and dried at 70 °C for overnight.

### Characterizations

X-ray diffraction patterns (XRDs) were collected by using powder XRD (Rigaku D/MAX-2500 X-ray diffractometer, with Cu Kα_1_ radiation λ = 0.154056 nm 40 kV and 40 mA, scanning from 10° to 70°). The microtopography of the synthesized materials was recorded with scanning electron microscopy (SEM, JSM-6700F) and high-resolution transmission electron microscopy (HRTEM, JEM-2100F, Japan). The high-resolution TEM and high-angle annular dark-field scanning TEM (HAADF-STEM) images were recorded on an FEI Tecnai G2 F20 S-Twin high-resolution transmission electron microscope (HRTEM; Hillsboro, OR, United States) set at 200 kV, and a JEOL JEM-ARM300F TEM/STEM (Tokyo, Japan), respectively, with a spherical aberration corrector worked at 300 kV. Through-focal HAADF series were acquired at nanometer intervals, with the first image under-focused (beyond the beam exit surface) and the final image over-focused (before the beam entrance surface). Then, the images were aligned manually to remove the sample drift effects. Electron paramagnetic resonance (EPR) spectra were recorded on a Bruker CW Elexsys E500 spectrometer by applying an X-band (9.4 GHz) microwave with sweeping magnetic field at room temperature.

## Results and Discussion

### Construction and Characterization of Pt_1_-MoS_2_ and Pt_1_-MoS_2_-def

The MoS_2_ were firstly synthesized by an anion-exchange strategy by using MoO_3_-ANE (aniline-functional MoO_3_) as precursors (Fig. S1). The SEM, TEM and HRTEM images in Fig. S2 reveal a uniform hierarchical nanosheet configuration with a well-ordered lattice fringe spacing of 0.27 nm, which agrees well with the (100) facet of hexagonal molybdenum disulfide. Subsequently, the Pt species were introduced through a deposition–precipitation process. As seen in Fig. S3, the high-angle annular dark-field scanning transmission electron microscopy (HAADF-STEM) image shows the atomic dispersion of Pt species over the Pt_1_-MoS_2_ surface and no notable Pt clusters are observed. Driven by thermal treatment of Pt_1_-MoS_2_ in a reductive atmosphere, Pt_1_-MoS_2_-def catalyst was synthesized. No remarkable architectonic changes and Pt nanoparticles are found (Fig. S4a, b). Meanwhile, the energy-dispersive X-ray spectroscopy (EDS) mapping (Fig. S4c) further demonstrates the homogeneous distribution of Pt, Mo and S elements. Furthermore, HAADF-STEM (Fig. 2a) clearly shows that the individual Pt atoms atomically disperse over the whole support surface without agglomerate, which is further confirmed by the extractive X–Y intensity profiles and atom-overlapping Gaussian-function fitting images of selective area scan (Fig. 2b–d). The simulating configuration (Fig. 2f) clearly exhibits the distribution of single dispersed Pt atoms, it is in accordance with the STEM image (Fig. 2e). For comparison, Pt nanoparticle modified MoS_2_ (Pt NPs-MoS_2_) was also synthesized through the ascorbic acid reduction in Fig. S5. More representative SEM, TEM, XRD and BET images of Pt_1_-MoS_2_, Pt_1_-MoS_2_-def, Pt NPs-MoS_2_ are displayed in Figs. S6-S9. Moreover, the Pt loading contents of Pt_1_-MoS_2_-def and Pt NPs-MoS_2_ are measured as 0.1% by inductively coupled plasma optical emission spectrometry (ICP-OES) (Table S1).

Element-selective X-ray absorption fine structure (XAFS) was further performed to explore the coordination environment of Pt species. As seen in Fig. 2g, the X-ray absorption near edge structure (XANES) spectra reveal the white line intensities of Pt_1_-MoS_2_-def and Pt_1_-MoS_2_ peaks are located between the PtS_2_ and Pt foil, suggesting the oxidation state of Pt^δ+^ (0 < *δ* < 4). The Fourier transferred extended X-ray absorption fine structure (FT-EXAFS) in Fig. 2h displays two notable peaks at ~ 1.90 Å, which are contributed by the Pt–S coordination path. No Pt–Pt characteristic peak (at ~ 2.57 Å) is detected for both Pt_1_-MoS_2_-def and Pt_1_-MoS_2_ samples, confirming the atomic dispersion of Pt on the supports. Moreover, the first-shell EXAFS fitting curves (Fig. 2i) and corresponding fitting results (Fig. S10 and Table S2) validate that the central Pt atom in both Pt1-MoS2 and Pt1-MoS2-def samples directly coordinates with three S atoms with the average bond lengths of 2.32 and 2.33 Å, respectively. Moreover, the wavelet transforms spectra of Pt EXAFS oscillations in Fig. 2j display only one peak at 7 Å^−1^ for Pt_1_-MoS_2_-def and Pt_1_-MoS_2_ samples, regarded as the Pt–S coordination contribution, indicating the high-distributed status of single Pt species.

### Feedback-regulation System of Pt_1_-MoS_2_-def

**Fig. 3:**
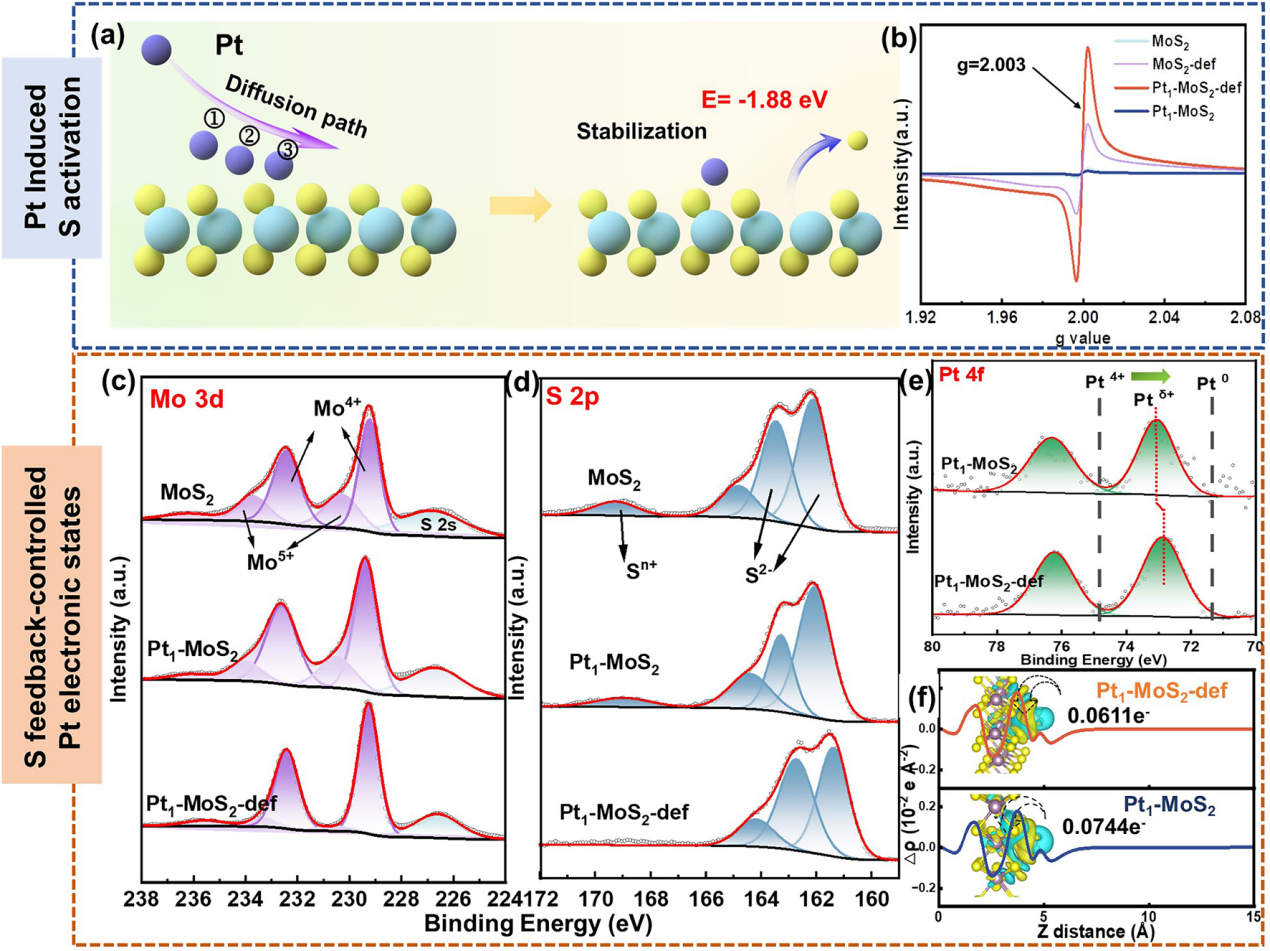
Structural characterizations of Pt_1_-MoS_2_-def and reference materials. **a** The diffusion path of Pt atoms on MoS_2_-def surface and diagram of S vacancy formation energy. **b** Electron-paramagnetic resonance images of MoS_2_, MoS_2_-def, Pt_1_-MoS_2_ and Pt_1_-MoS_2_-def. **c** Mo 3*d* XPS of MoS_2,_ Pt_1_-MoS_2_ and Pt_1_-MoS_2_-def. **d** S 2*p* XPS of MoS_2,_ Pt_1_-MoS_2_ and Pt_1_-MoS_2_-def. **e** Pt 4*f* XPS of Pt_1_-MoS_2_ and Pt_1_-MoS_2_-def. **f** The planar-averaged electron density difference ∆*ρ*(*z*) of Pt_1_-MoS_2_ and Pt_1_-MoS_2_-def

The first-principal calculations were further conducted to verify the Pt-site configuration on the support. Figure 3a simulates the diffusion path of the Pt atom on the MoS_2_ surface, including ①Pt on the S site, ②Pt on the hollow site and ③Pt on the Mo site. The results of the DFT calculation (Fig. S11) show that the Pt atom can stabilize on the top of the Mo site with the lowest binding energy of − 3.265 eV in contrast to − 2.764 eV (Pt on the S site) and 6.024 eV (Pt on the hollow site). Also, this configuration of Pt atom bonding with three S atoms is consistent with the XAFS fitting results. After further treating the Pt_1_-MoS_2_ in a reduced atmosphere, the S atoms will spontaneously escape from the MoS_2_ surface to generate sulfur vacancy sites (Pt_1_-MoS_2_-def). This process will induce five potential configurations in Fig. S12. Considering the adjudication of experimental investigations (detailed descriptions in supporting information), the defect 2 model (adjacent non-bonding S defect) was selected as the optimal configuration with the lowest vacancy formation energy of − 1.876 eV. This spontaneous reaction process (Fig. S13) indicates that the introduced single Pt atom can promote the evaporation of adjacent S species. Importantly, this three-coordinated structure of Pt_1_-MoS_2_-def is also well conforming by XAFS results. Furthermore, the theoretical deduction can be verified by the experimental electron paramagnetic resonance (EPR) analysis. As shown in (Fig. 3b), the signal intensity of sulfur vacancies (at *g* = 2.003) of Pt_1_-MoS_2_-def is significantly higher than that of MoS_2_-def [29] (more synthetic process showed in supporting information), demonstrating single Pt atoms can induce the formation of sulfur vacancies and thus activate the whole S plane.

The XPS measurements are employed to unveil the chemical environment of Mo (Fig. 3c), S (Fig. 3d) and Pt (Fig. 3e) species. As shown in Fig. 3c, the peaks at 231.9 eV (Mo 3*d*_3/2_) and 228.7 eV (Mo 3*d*_1/2_) are attributed to Mo^4+^ and the peaks at 233.9 eV (Mo 3*d*_3/2_) and 230.7 eV (Mo 3*d*_1/2_) are assigned to Mo^5+^ [30], respectively. After introducing the Pt species, the doublet peaks of both Mo^4+^ (232.1 and 228.9 eV) and S^2−^ (161.9 and 163.2 eV) in the Pt_1_-MoS_2_ shift to higher binding energy (0.2 and 0.3 eV left shifts, respectively) compared with pure MoS_2_. Meanwhile, the binding energy of Pt 4*f* peak is higher than Pt^0^ (71.2 eV, Fig. S14) but lower than that of Pt^4+^ (74.0 eV) [26] according to the XAFS results. For Pt_1_-MoS_2_-def catalysts, the double peaks of Mo^5+^ (233.9 and 230.7 eV) and the high valence S^*n*+^ peaks (164.5 and 169.2 eV) gradually disappear. The Pt peaks in Pt_1_-MoS_2_-def (72.8 eV) shift toward lower binding energy (0.3 eV) in contrast to the Pt_1_-MoS_2_ sample. The theoretical planar-averaged electron density difference ∆*ρ*(*z*) and Bader charge transfer in Fig. 3f further verify that the Pt atom in the as-established Pt_1_-MoS_2_-def model can reduce the electronic loss toward supports, according well to experimental XPS investigation. Both experimental and theoretical results reveal that activated S species will provide a feedback role in tailoring the electronic states of Pt atoms. In conclusion, the single Pt sites on the MoS_2_ surface can induce easier volatilization of adjacent S species and the activated S species can inversely impact the electronic configuration of Pt atoms, thus leading an intact feedback-regulation system.

### Sensing Properties and Mechanism of MoS_2_, Pt_1_-MoS_2_ and Pt_1_-MoS_2_-def

**Fig. 4:**
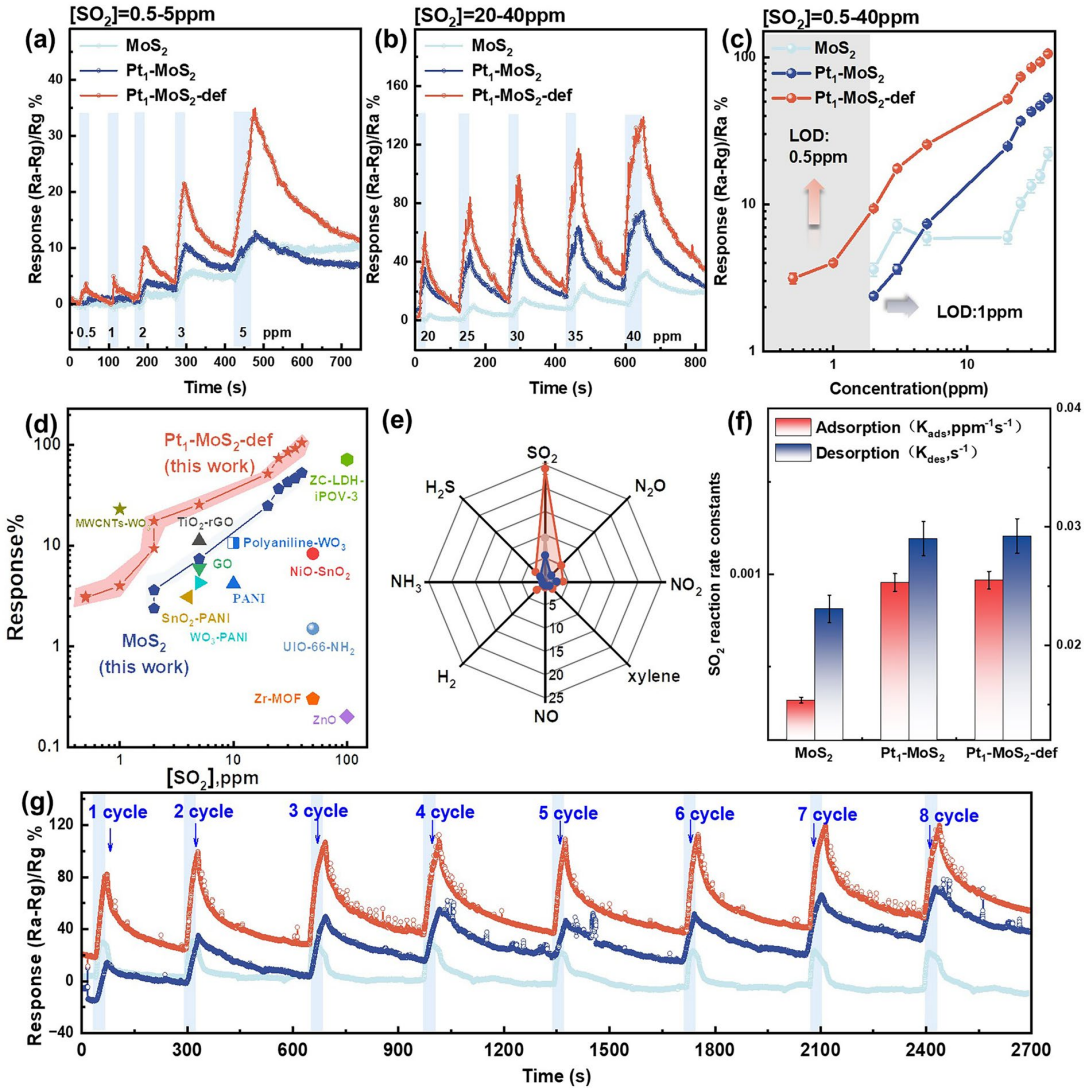
Gas-sensing performance of MoS_2_, Pt_1_-MoS_2_ and Pt_1_-MoS_2_-def. **a, b** Time-related dynamic responses in the concentration range of 0.5–5 and 20–40 ppm. **c** The responses of MoS_2_, Pt_1_-MoS_2_ and Pt_1_-MoS_2_-def in different SO_2_ concentrations. **d** RT operation state-of-the-art SO_2_ chemiresistors for response. **e** The selectivity of MoS_2_, Pt_1_-MoS_2_ and Pt_1_-MoS_2_-def in different gases. **f** Adsorption and desorption rate constant *K*_ads_ and *K*_des_ values. **g** stability tests using sensors upon 8 cyclic exposures to 25 ppm SO_2_. (All the sensing tests were conducted at RT in (30%RH) condition)

The sensing properties of the MoS_2_, Pt_1_-MoS_2_ and Pt_1_-MoS_2_-def MEMS sensors were investigated at room temperature (RT). The dynamic response transitions curves of the sensors along with SO_2_ concentrations in the range of 0.5–40 ppm are exhibited in Fig. 4a, b. Obviously, the Pt_1_-MoS_2_-def sensor displays the highest response values toward different concentrations of SO_2_ compared with Pt_1_-MoS_2_ and MoS_2_ sensors. Some baseline drift of the three sensors at room temperature could be attributed to the incomplete desorption of the SO_2_ molecule [31]. Especially, the Pt_1_-MoS_2_-def sensors (25% to 5 ppm SO_2_) exhibit an enhanced response approximately four times and five times higher than that of Pt_1_-MoS_2_ (7% to 5 ppm SO_2_) sensors and MoS_2_ sensors (5% to 5 ppm SO_2_), respectively. Figure 4c shows the linear relationships between responses and SO_2_ concentrations within 0.5–40 ppm for three sensors. Impressively, the Pt_1_-MoS_2_-def sensors exhibit an exceptional linear section with increased SO_2_ concentrations, while the MoS_2_ sensors endure the erratic response-concentration relationship. Additionally, the experimental limit of detection (LOD) of the Pt_1_-MoS_2_-def sensor can reach as low as 500 ppb, whereas the Pt_1_-MoS_2_ sensors and MoS_2_ sensors cannot perceive SO_2_ gas below 1 ppm. Such a distinguished response performance exceeds that of Pt NPs-MoS_2_ sensors (Fig. S15) and most reported SO_2_ sensors (Fig. 4d and Table S4). More dynamic response-recovery resistance curves of MoS_2_, Pt_1_-MoS_2_ and Pt_1_-MoS_2_-def sensors are shown in Fig. S16. As shown in Fig. 4e, eight kinds of typical vaporous molecules including sulfur dioxide (SO_2_), ammonia (NH_3_), hydrogen (H_2_), sulfuretted hydrogen (H_2_S), nitric oxide (NO), xylene (C_8_H_10_), nitrogen dioxide (NO_2_) and nitrous oxide (N_2_O) were detected at the same concentration of 5 ppm at RT. It is found that the responses of interfering gases are significantly negligible. Furthermore, the calculated selectivity coefficients (*K*) of all sensors (Fig. S17) reveal relatively high *K* values, indicating excellent SO_2_ selectivity for gas-sensing performances. The adsorption (*k*_ads_) and desorption (*k*_des_) rate constants of all sensors (Fig. 4f) were further calculated by fitting the response versus time curves (Fig. S18) using the equations below:

1 $$R\left(t\right) \text{ for} \, {\text{SO}}_{2} \, {\text{adsorption}} \text{ = }{R}_{{\text{max}}\bullet }\frac{{C}_{a}K}{1+{KC}_{a}}\left(1-{e}^{\left[-\frac{1-{KC}_{g}}{K}\bullet {k}_{ads}t\right]}\right)$$

2 $$R\left(t\right) \text{ for} \, {\text{SO}}_{2} \, \text{desorption } = {R}_{0}{e}^{\left[-{k}_{des}t\right]}$$

where *R*_max_ is the maximum response value, *R*_0_ is the response in air, *C*_*a*_ is the concentration of SO_2_ gas, *t* is the time and *K* is the equilibrium constant (*k*_ads_/*k*_des_) [32]. The results indicate that the kinetic adsorption and desorption of SO_2_ molecules are promoted by Pt species and sulfur vacancies. In addition, the cycle stability (Fig. 4g) and long-term durability measurements (Fig. S19) show that the Pt_1_-MoS_2_-def sensor can remain almost unchanged responses during continuous eight-cyclical exposure of SO_2_ and superior stability for up to 35 days. The effects of operating temperature for Pt_1_-MoS_2_-def sensor were conducted in Fig. S21, in which the sensors can realize optimal gas-sensing performance at room-temperature conditions. Besides, the resistances of the Pt_1_-MoS_2_-def sensor may be affected by the humidity environment (Fig. S22), which is mainly because water molecules might react with SO_2_ molecules. A water filtration membrane to filter out water molecules was added on the device (Fig. S23a). As shown in Fig. S23b, c, after adjusting the device, the response of the material to 25 ppm SO_2_ under different humidity conditions were tested. It was observed that under 90% humidity, the sensor’s response decreased by no more than 9% (before the improvement, the sensor’s response decreased by about 28%), effectively improving the normal operation of the device in high humidity environments.

**Fig. 5:**
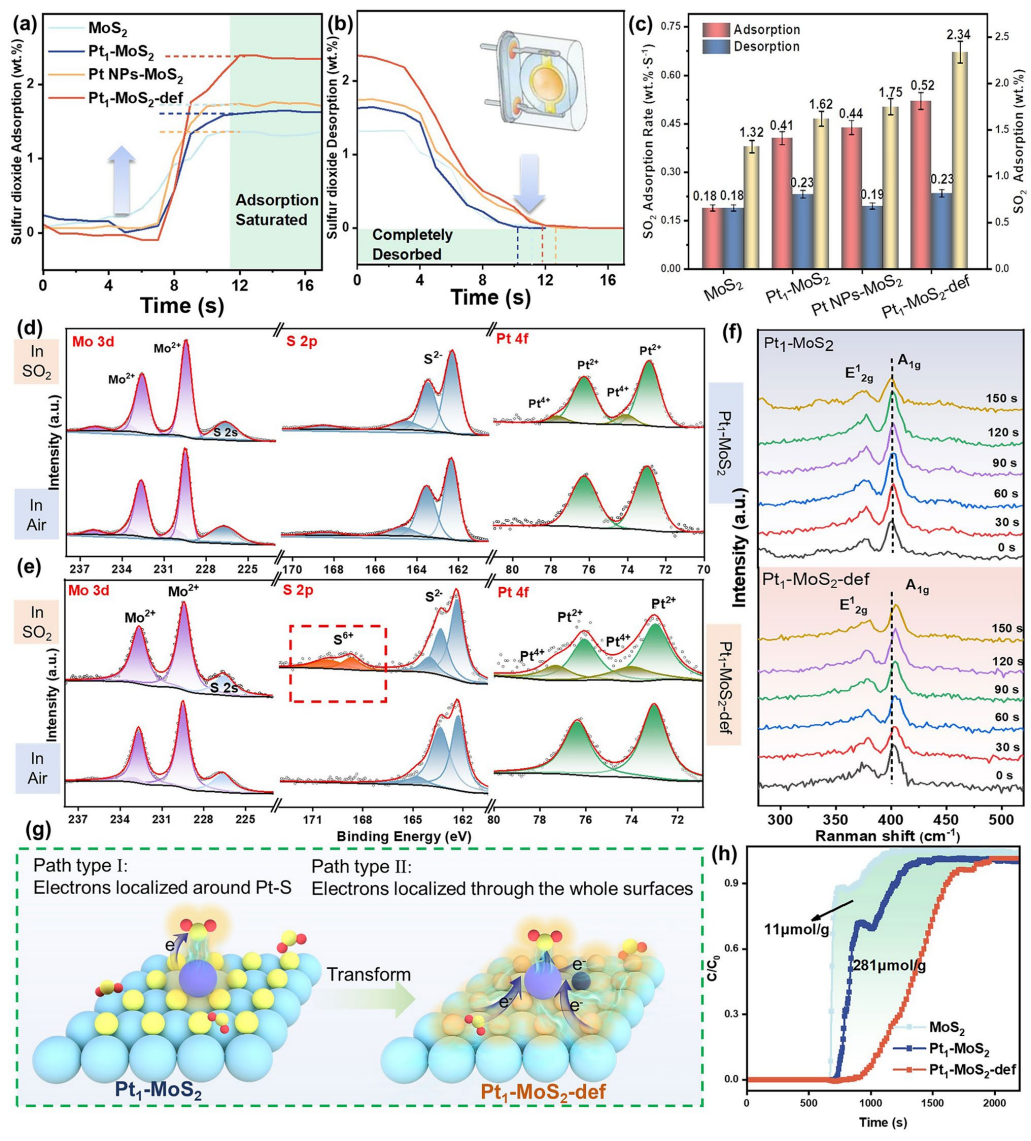
The experimental investigation of the mechanism for the gas-sensing property of Pt_1_-MoS_2_-def. **a** The SO_2_ adsorption curves, **b** desorption curves and **c** SO_2_ adsorption rate of MoS_2_, Pt_1_-MoS_2_, Pt NPs-MoS_2_ and Pt_1_-MoS_2_-def. The ex situ XPS spectra of **d** Pt_1_-MoS_2_ and **e** Pt_1_-MoS_2_-def. **f** In situ Raman spectra of the Pt_1_-MoS_2_ and Pt_1_-MoS_2_-def. **g** Two different path types of Pt_1_-MoS_2_ and Pt_1_-MoS_2_-def with SO_2_. **h** In-situ SO_2_ adsorption breakthrough curves of MoS_2_, Pt_1_-MoS_2_ and Pt_1_-MoS_2_-def at 25 °C

To further clear the design superiority of the Pt_1_-MoS_2_-def catalysts in the gas-sensing reaction, SO_2_ adsorption and desorption tests were implemented (experimental details are shown in Methods and Table S3). As Fig. 5a–c shows, the Pt_1_-MoS_2_-def reveals the maximum adsorption capacity (2.34 wt%) and the fastest average adsorption rate (0.52 wt% s^−1^) for SO_2_ compared with MoS_2_ (1.32 wt%, 0.18 wt% s^−1^), Pt_1_-MoS_2_ (1.62 wt%, 0.41 wt% s^−1^) and Pt NPs-MoS_2_ (1.75 wt%, 0.44 wt% s^−1^) at room temperature, indicating the S vacancy-assisted single Pt sites (Pt-Vs) can induce superior adsorption and excitation ability for sulfur dioxide. Ex situ XPS spectra were further employed to investigate the reaction mechanism of different catalysts. As seen in Fig. 5d, the peaks of Pt^4+^ in Pt_1_-MoS_2_ suddenly appear in the SO_2_ atmosphere while no obvious new peaks are found in the Mo and S species, confirming the electron transfer path is from the single Pt site to adsorbed SO_2_ molecule. However, a small number of surface-adsorbed SO_2_ molecules are not distinguished by the XPS detection. Same results can also be found in MoS_2_-def catalysts in Fig. S24. Considering the resistances of MoS_2_-based sensors significantly reduce in the oxidizing SO_2_ atmosphere (Fig. S25), the sensing type can be identified as p-type semiconductors. Detailly, the SO_2_ species can capture electrons from the conduction band of p-type semiconductors and induce the hole generation, resulting in decreased resistances (Fig. S26). For the Pt_1_-MoS_2_-def catalyst (Fig. 5e), two new higher-valence S peaks (168.83 and 170.1 eV, S^6+^) and Pt^4+^ peaks are discovered after SO_2_ treatment while there is a slight high binding energy shift in the Mo 3*d* spectrum, revealing that both Pt and MoS_2_ supports lose electrons during the gas-sensing process. The in situ Raman spectra display the 150 s exposure periods of Pt_1_-MoS_2_-def, Pt_1_-MoS_2_ (Fig. 5f) and MoS_2_-def (Fig. S27) after treating with 1000 ppm SO_2_. Clearly, the A_1g_ peak (at 402.25 cm^−1^) of the Pt_1_-MoS_2_-def sample shifts to a higher wavenumber after SO_2_ exposure for 60 s, while no peak changes for the Pt_1_-MoS_2_ and MoS_2_-def samples. Therefore, this unequivocally corroborates that the electronic transform process in Pt_1_-MoS_2_-def involves the adsorbed SO_2_ molecules and the whole supports. In conclusion, experimental results demonstrate there are two potential reaction paths dominant to the SO_2_ sensing performance for Pt_1_-MoS_2_-def and Pt_1_-MoS_2_ sensors. For Pt_1_-MoS_2_ (Fig. 5g, type I), the electrons mainly locate around the single Pt site and the electron transfer only occurs within Pt–S (SO_2_). In comparison, the Pt_1_-MoS_2_-def sensors contain synergistically single Pt sites and activated inert S plane, which prompts the whole supporting surface to participate in the SO_2_ sensing process (Fig. 5g, type II). Meanwhile, the rapid electron transfer between Pt_1_-MoS_2_-def and the oxidizing SO_2_ guarantees high response and low LOD. In addition, the in situ SO_2_ adsorption breakthrough curves (Fig. 5h) were implemented to quantitatively measure the absorption of SO_2_, in which the adsorbed SO_2_ amounts on Pt_1_-MoS_2_-def is 281 μmol g^−1^ more than that on Pt_1_-MoS_2_, and there is 292 μmol g^−1^ more than that on MoS_2_-def at 25 °C, according well with the SO_2_ adsorption and desorption tests in Fig. 5a–c.

### Theoretical Investigations

**Fig. 6:**
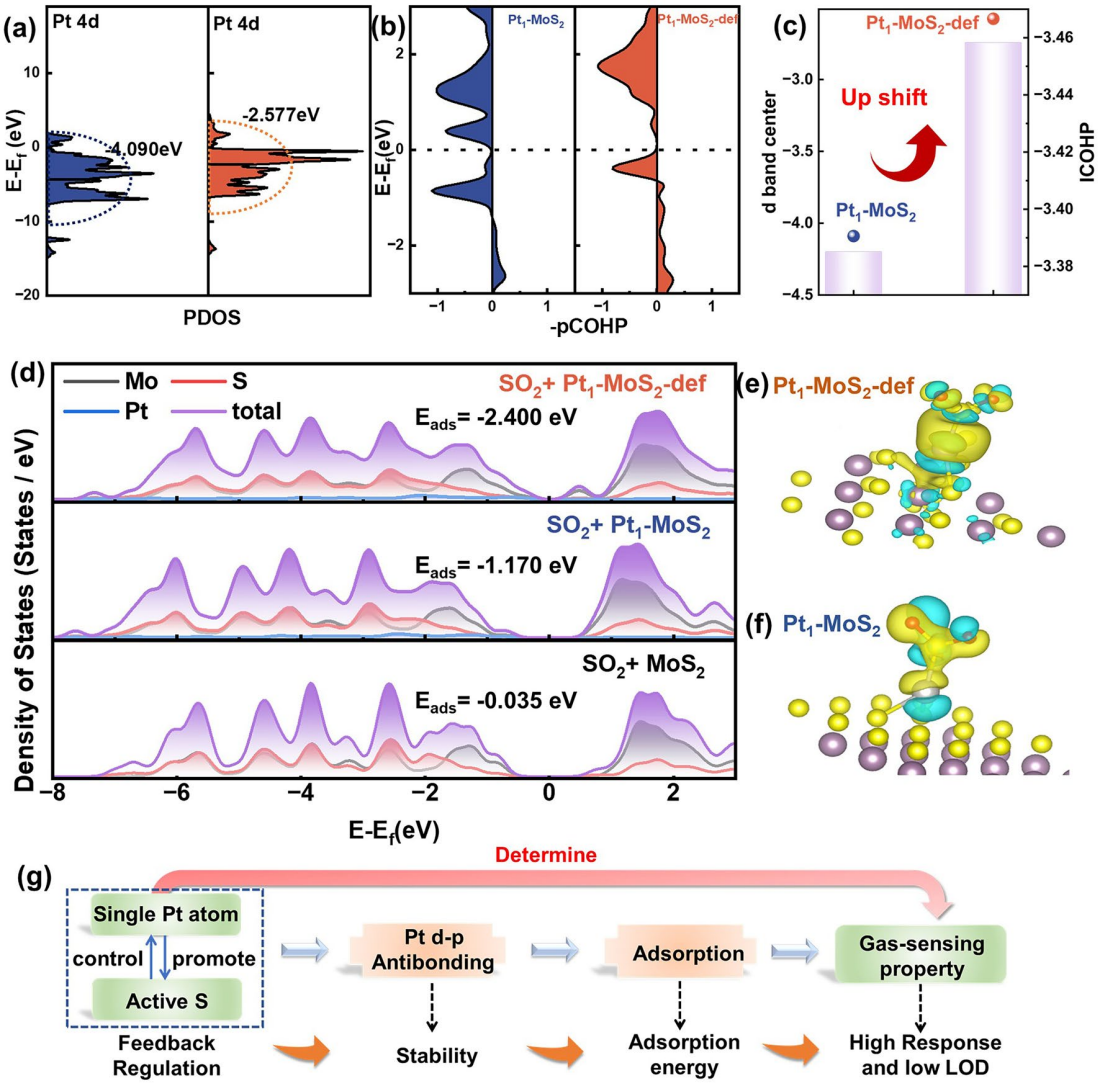
The theoretical investigation of the mechanism for the gas-sensing property of Pt_1_-MoS_2_-def. **a** PDOS for Pt 4*d* orbital and the d band center of Pt_1_-MoS_2_ and Pt_1_-MoS_2_-def. **b** pCOHP and **c** ICOHP of terminal Pt–S bonds of Pt_1_-MoS_2_ and Pt_1_-MoS_2_-def. **d** The partial density of states (pDOS) analysis and adsorption energy of SO_2_/Pt_1_-MoS_2_-def, SO_2_/Pt_1_-MoS_2_ and SO_2_/MoS_2_ models. The electronic transfer between SO_2_ and **e** Pt_1_-MoS_2_-def and **f** Pt_1_-MoS_2_ sample. The yellow and blue lobes represent the accumulation and depletion of charge. **g** Mechanism for tailoring the level of antibonding-orbital occupancy state to unidirectionally determined the gas-sensing property of SO_2_

DFT calculations were conducted to explore the structure–activity relationships between single Pt sites and gas-sensing performance. As seen in Fig. 6a, the Pt 4*d* partial DOS (pDOS) results show that the d-band center in Pt_1_-MoS_2_-def (-2.58 eV) obviously upshifts compared with the Pt_1_-MoS_2_ (− 4.10 eV). The corresponding DOS images are displayed in Fig. S28. When interacting with SO_2_ molecule, the Pt–S (SO_2_) projects crystal orbital Hamilton population (pCOHP) is calculated in Fig. 6b, where the negative –pCOHP represents the antibonding contribution and the positive –COHP stands for bonding contribution [33–35]. Obviously, the antibonding contribution gradually moves upward (compared to *E*_*f*_) from Pt_1_-MoS_2_-def to Pt_1_-MoS_2_, proving a decreased occupation state and a strengthening Pt–S bond. The –IpCOHP (the integral of –COHP up to the Fermi level) values are further calculated in Fig. 6c. The antibonding orbital occupation state of the Pt–S bond in Pt_1_-MoS_2_-def is smaller than that in Pt_1_-MoS_2_, indicating more stable adsorption of SO_2_ for Pt_1_-MoS_2_-def, which is conformed to the d-band center theory. In addition, the density of states (DOS) analysis in Fig. 6d displays that a new peak around Fermi energy level could be observed in SO_2_/Pt_1_-MoS_2_-def, suggesting more electrons transfer between SO_2_ and Pt_1_-MoS_2_-def compared with SO_2_/Pt_1_-MoS_2_ and SO_2_/MoS_2_ models. This can be further verified by the differential charge density comparisons in Fig. 6e, f. Also, the Pt_1_-MoS_2_-def reveals larger the adsorption energy (*E*_ads_) of -2.40 eV than that of the Pt_1_-MoS_2_ (− 1.17 eV) and MoS_2_ (− 0.035 eV) upon interaction with SO_2_ molecules (Fig. S29), according well with gas-sensing performance. Overall, utilizing Pt species to promote adjacent S evaporation, the electronic state of S vacancy-assisted single Pt atom pronouncedly changes and thus induces a gradually decreased antibonding contribution state of Pt–S and a stronger SO_2_ adsorption. Hence, the establish of feedback-regulation system is crucial for SO_2_-sensing material with high response and low LOD (Fig. 6g).

### Bluetooth-Based Devices for Monitoring the Environment of Plant Growth

**Fig. 7:**
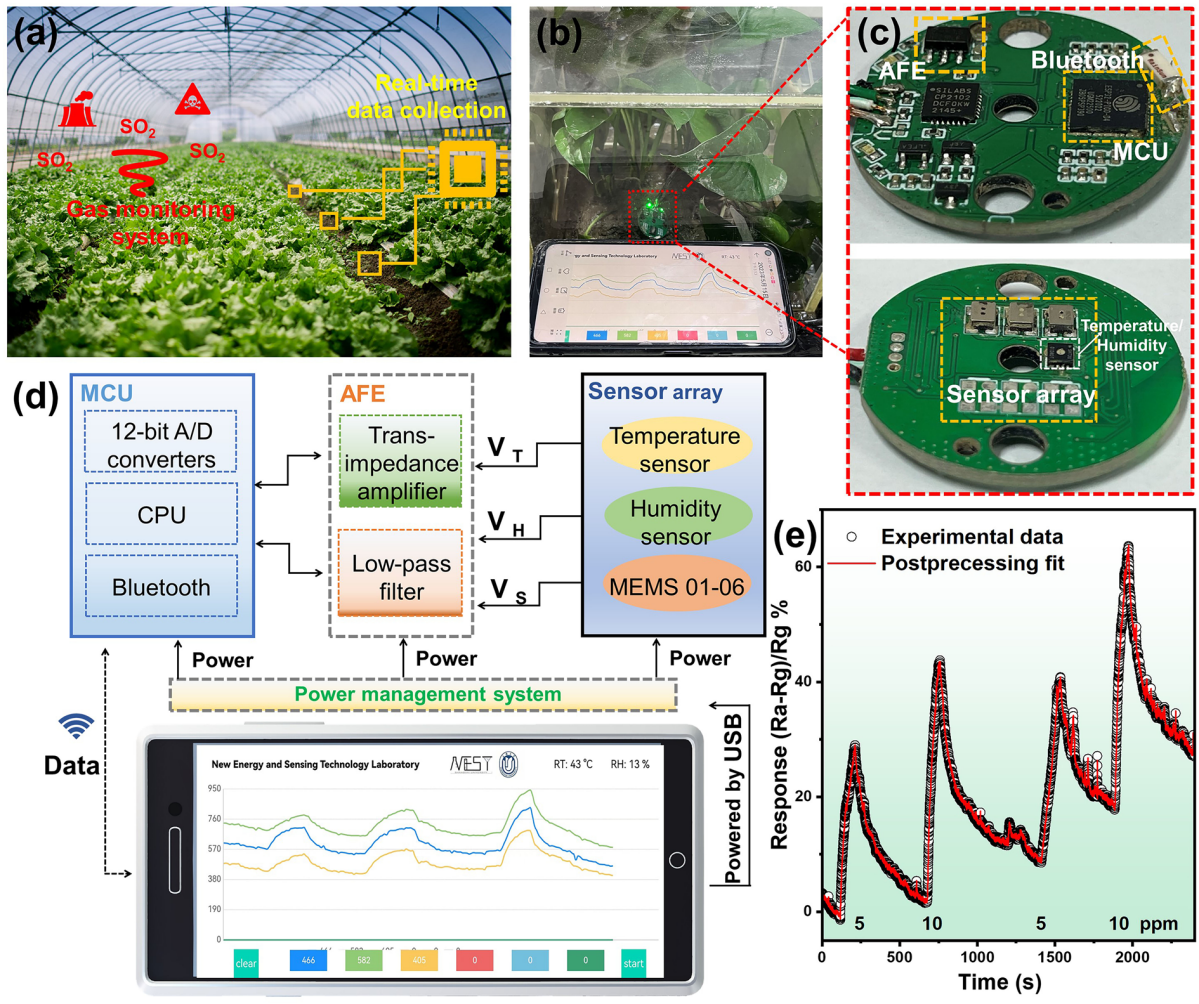
Pt_1_-MoS_2_-def-based SO_2_ gas sensors for monitoring the plants growth. **a** Schematic of real-time detection of SO_2_ gas concentration in a greenhouse. **b** Photo of the device in a simulated greenhouse with plants, with a smartphone and Bluetooth and data transmission. **c** Corresponding block modules. **d** Block diagram of the whole system. **e** Data measured by this device

As we all known, the real-time monitor, track and prediction of stressful circumstances (such as SO_2_) is of vital importance to optimize and adjust plant growth [8]. For instance, an appropriate SO_2_ environment can promote seed germination and stomatal movement, while excessive SO_2_ concentrations will disturb the physiological and biochemical metabolism [36–38]. Therefore, engineering multifunctional SO_2_ sensors array to timely collect and transmit the gas/temperature/humidity signals is desired for plant cultivation process (Fig. 7a). Based on the superior SO_2_-sensing materials of Pt_1_-MoS_2_-def, a miniaturized, integrated and wireless sensing SO_2_ sensors device is further developed in Fig. 7b, c. The disk-shaped device is composed of Bluetooth, Active Front End (AFE) and Micro Controller Unit (MCU) on one side, while a MEMS array (containing six sensors) and temperature/humidity modules are embedded at the back. Wireless data transmission from devices to smartphones can be achieved through the Bluetooth system. The corresponding block diagram is presented in Fig. 7d, including the power delivery, data conditioning and data transmission pathways. Impressively, synchronous six sensing signals can be received and revealed on the screen to calibrate the errors. More details of printed circuit board and the main interfaces of the custom mobile application device are shown in Figs. S30–S33. Furthermore, the portable sensing device displays constant responses for 5 and 10 ppm SO_2_ gases (Fig. 7e) in a realistic greenhouse environment. More gas-sensing performance tests are shown in Fig. S34. Notably, the current design still stays in a proof-of-concept stage, it may require more optimizing process including signal handling, data integration, intellectual detection and so on in future work.

## Conclusions

In summary, by designing the feedback-regulation system in Pt_1_-MoS_2_-def sensing materials, the superior SO_2_ wireless detection sensors can be realized_._ When introducing the single Pt atom to MoS_2_ surface, the adjacent S species is inclined to evaporate to form S vacancy-assisted single Pt sites. Reversely, the electronic state of Pt atom pronouncedly changes by the activated S planes. After treating with SO_2_ gas, the antibonding contribution states of Pt–S gradually decreased and thus induce a stronger SO_2_ adsorption. This result can further be confirmed by the gas adsorption/desorption experiments and in situ gas adsorption breakthrough experiments. The final Pt_1_-MoS_2_-def sensors show extremely high performance for low-concentration SO_2_ gas sensing at room temperature (3.14% to 500 ppb). Combined with a Bluetooth transmission system, the Pt_1_-MoS_2_-def sensor arrays can further achieve real-time SO_2_ monitoring and data transmission under real conditions for plant growth. Our work is thus expected to broaden the rational design of highly effective gas sensors.

## Supplementary Information

Below is the link to the electronic supplementary material.

Supplementary file1 (PDF 4520 KB)

Supplementary file2 (PDF 83694 KB)
