# Supplementary material for: Building Feedback-Regulation System Through Atomic Design for Highly Active SO2 Sensing
Source: Nanomicro Lett. 2024 Feb 27;16:136. doi: 10.1007/s40820-024-01350-3 (PMC10899126; doi:10.1007/s40820-024-01350-3)
Supplement: Supplementary file 1 — Supplementary file1 (PDF 4520 KB) [file 40820_2024_1350_MOESM1_ESM.pdf]

Nano-Micro Letters

Supporting Information for

Building Feedback-Regulation System through Atomic Design for Highly Active SO_2 Sensing

Xin Jia[1,†], Panzhe Qiao[2,†], Xiaowu Wang[1], Muyu Yan[3], Yang Chen[1,3], Bao-Li An[1], Pengfei Hu[5], Bo Lu[5], Jing Xu[5], Zhenggang Xue[1,*], Jiaqiang Xu[1,*]

[1]NEST Lab, Department of Chemistry, College of Sciences, Shanghai University, Shanghai 200444, P. R. China

[2]Shanghai Synchrotron Radiation Facility, Zhangjiang Lab, Shanghai Advanced Research Institute, Chinese Academy of Sciences, Shanghai 201204, P. R. China

[3]School of Chemistry and Materials Science, University of Science and Technology of China, Hefei 230026, P. R. China

[4]Key Laboratory of Organic Compound Pollution Control Engineering (MOE), School of Environmental and Chemical Engineering, Shanghai University, Shanghai 200444, P. R. China

[5]Shanghai University, Instrumental Analysis & Research Center of Shanghai University, Shanghai 200444, P. R. China

[†]Xin Jia and Panzhe Qiao contributed equally to this work.

[*]Corresponding authors. E-mail: zgxue@shu.edu.cn (Zhenggang Xue); xujiaqiang@shu.edu.cn (Jiaqiang Xu)

S1 Experiment Detail

S1.1 XAFS measurements

The X-ray absorption fine structure data were collected at 1W1B station in Beijing Synchrotron Radiation Facility (BSRF). The storage rings of BSRF was operated at 2.5 GeV with a maximum current of 250 mA. The X-ray absorption near edge structure (XANES) data were recorded in fluorescence mode. All samples were pelletized as disks of 13 mm diameter using poly(1,1-difluoroethylene) powder as a binder. The acquired extended X-ray absorption fine structure (EXAFS) data were processed according to the standard procedures using the ATHENA module implemented in the IFEFFIT software packages [S1]. The EXAFS spectra were obtained by subtracting the post-edge background from the overall absorption and then normalizing with respect to the edge-jump step. Subsequently, $\chi(k)$ data in the k-space were Fourier transformed to real (R) space using a Hanning window (dk = 1.0 $Å^{-1}$) to separate the EXAFS contributions from different coordination shells.

S1.2 *Ex situ* XPS experiments

Ex situ XPS measurements were carried out on X-ray photoelectron spectroscopy (XPS, ESCALAB 250Xi, Thermofisher Scientific) and binding energies were calibrated the of the elements by C 1s (284.6 eV). During the test, the sample was first mounted on a transfer bar and then transferred to the reaction chamber. Next, a gold-plated copper seal was installed to seal the reaction chamber. Then, the target temperature (25°C) is set and the corresponding air is injected for pretreatment for 5 minutes. Finally, the reaction chamber is evacuated, and then transferred directly to the analysis chamber for testing and analysis. The data obtained is the XPS spectra in air. The reaction chamber was transferred out of the analysis chamber. The corresponding mixture of SO_2 gas and air is injected for pretreatment for 5 minutes (SO_2 in the gas mixture at a

concentration of 50 ppm). Then, the reaction chamber was vacuumed, and then directly transferred to the reaction chamber for testing and analysis. The obtained data is XPS spectra in SO_2.

S1.3 *In situ* gas (SO_2) adsorption breakthrough

In situ gas (SO_2) adsorption breakthrough tests combined with mass spectrometry (MS) were carried out using the dynamic sorption analyzer (mixSorb S) with a thermal conductivity detector (TCD) combined with a mass spectrometer (OMNISTARTM).

S1.4 *In situ* Raman experiments

The Raman spectra were obtained on a Renishaw spectrometer (Raman, INVIA). The sample was first placed on a silicon wafer in the in-situ reactor. Then a mixture of SO_2 gas and air was injected and the Raman spectrum is collected (1000 ppm of SO_2 in the gas mixture). Finally, data is collected every 30 s and the last data is collected at 150 s to end the test.

S1.5 Gas-sensing measurement

The gas-sensitive material was coated on the interdigital electrode of Micro Electro Mechanical System (MEMS) chip by ultrasonic spraying equipment which ensured the uniformity of the materials on the chips. As seen in Fig. S35, the thickness of the sensing layer was about 58 μm. Assembled MEMS sensors were placed onto the bottom of the chamber of LP-002A gas sensing system (Lingpan Electronics Technology Co. Ltd., China). The gas sensing system were shown in Fig. S36. The circuit voltage (V) was set at 10 V and the output voltage (Vout) was recorded 10 times a second. The relationship between the resistance of the MEMS sensor (R) and the Vout is shown in Equation S1. The load resistance (R_L) is 10^6 W in the gas sensitivity test. Therefore, R can be calculated by Vout. The resistance (R) of sensor were calculated by the Eq. (S1):

$$R = \left(\frac{10-V_{out}}{V_{out}}\right) \cdot R_L \qquad \text{(S1)}$$

Where 10 is the circuit voltage, V_{out} is the output voltage of the load resistance (R_L) detected. The operating temperature of sensors was achieved by adjusting the heater voltage. During the gas sensitivity testing process, a constant temperature (25℃) and humidity (30%-45%) are rigorously controlled in the testing laboratory, which ensuring that each gas sensitivity test was conducted under the same temperature and humidity conditions. All the different concentrations of standard gases are 200 ppm standard gases mixed with N_2 bought from Shanghai Shenkai Gases Technology Co., Ltd. The static gas distribution method was used for testing the gas response. When the resistances kept stable, the test gas was injected into the 200 mL chamber. The amount of injected gas can be obtained through the Eq. (S2):

$$C_x = \frac{V_x}{200} \cdot C \qquad \text{(S2)}$$

Where V_x(mL) is the gas volume extracted from the gas sampling bags by the micro syringe, 200 is the volume of the test chamber, C is the standard concentration of gas in gas sampling bags and the C_x is the different gas concentration in the test chamber. The response value of sensors in a reducing atmosphere is defined as response = $(R_a\text{-}R_g)/R_g$. The resistance of the sensor in the air is recorded as R_a and that in the test gas is R_g. The test temperature is changed by changing the voltage at both ends of the heating electrode. Figure S38 is the relationship between the voltage at both ends of the heating electrode and the temperature.

The selectivity coefficient (K) can be used to quantitatively assess the selectivity of the sensor. The K value can be calculated by the Eq. (S3):

$$K = \frac{response_{SO2}}{response_x} \qquad \text{(S3)}$$

where the response SO_2 is the response of the sensor in 5 ppm SO_2 and the response x is the response of the sensor in 5 ppm other gases.

S1.6 Gas (SO_2) Adsorption and Desorption Tests

The Quartz crystal microbalance (QCM) resonators were purchased from Chengdu West Sensor Technology Co., Ltd. QCM measurement is composed of a thin slice of quartz crystal with double-faced deposited silver electrodes. The mechanism of QCM is based on the piezoelectric effect of quartz crystal oscillator, where there is a quantitative relationship between the increasing mass change (Δm) and decreasing frequency change (Δf). The quantitative relationship is in accordance with the Sauerbrey equation: $\Delta f=-2.3\times10^{-6}\times f_0^2\Delta m/A$. Where f_0 is the inherent oscillation frequency of the chip (10^7, Hz), A is the effective working area of the electrodes (d=3 mm).

S1.7 Methodology and calculation model

All DFT computations were performed in the Vienna ab initio simulation package (VASP) [S2] under projector augmented wave (PAW) potentials to describe the interactions. The generalized gradient approximation (GGA) [S3] in the Perdew–Burke–Ernzerhof (PBE) functional was used to treat the exchange-correlation between electrons. The way of DFT-D3 [S4] was adopt to correct van der Waals force. A cut-off energy of 400 eV for plane wave expansions was used and all geometric structures were set a 10 Å vacuum layer in the z-direction. The 10^{-5} eV for conventional energy and 0.02 eV $Å^{-1}$ for force under a 3×3×1 sheet k-point mesh were taken as the convergence criteria to optimize the structures. The electronic structures of the density of state (DOS) were calculated with 3×3×1 k-points. In all of the structure optimization, the bottom half of the slab in the vertical z-direction was constrained, while the top half of the slab and the adsorbates were fully relaxed. In this calculation, the MoS_2(200) was modeled by a 2×2 supercell. The adsorption energy (ΔE) of SO_2 on the substrates can be given by Eq. (S4):

$$\Delta E = E_{total} - E_{SO2} - E_{substrate} \quad \text{(S4)}$$

Where E_{total}, E_{SO2}, and $E_{substrate}$ are the energy of SO_2 adsorption on the substrate, energy of SO_2, and energy of substrate, respectively.

S2 Supporting Figures and Tables

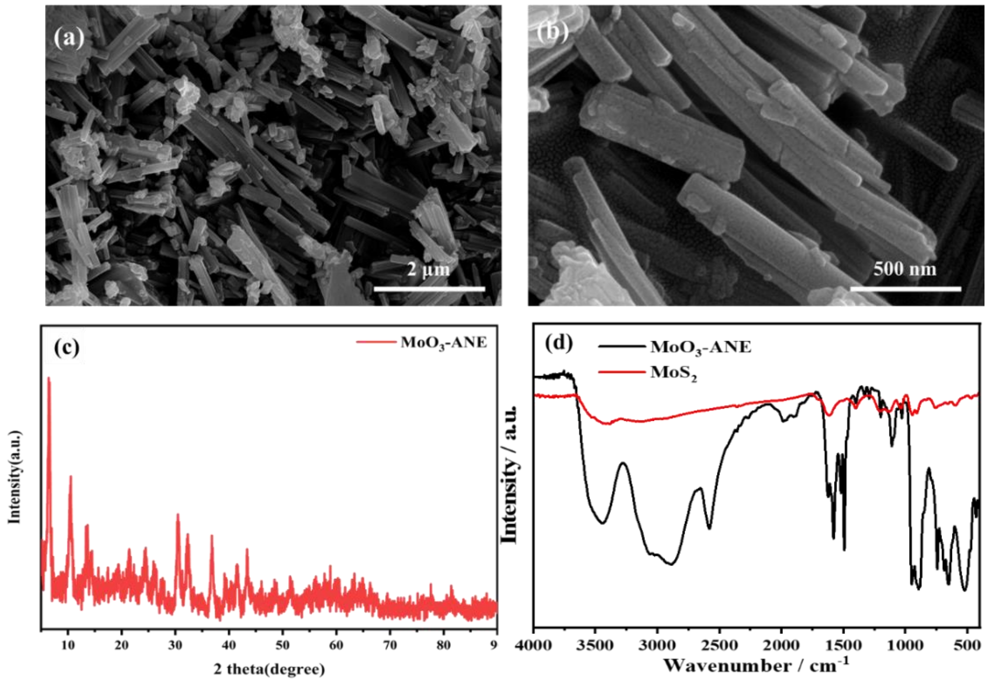

Fig. S1 (**a, b**) SEM images of MoO_3 inorganic-organic nanowires. (**c**) XRD pattern of the MoO_3-ANE (**d**) FTIR spectra of the MoO_3-ANE inorganic-organic nanowires and MoS_2

All the diffraction peaks of MoO_3-ANE were well indexed to monoclinic $Mo_3O_{10}(C_6H_8N)_2$ (ICDD: 50-2402), thus matching those reported previously [S5]. The FTIR spectra suggest that the hybrid precursors can be successfully transformed into inorganic materials.

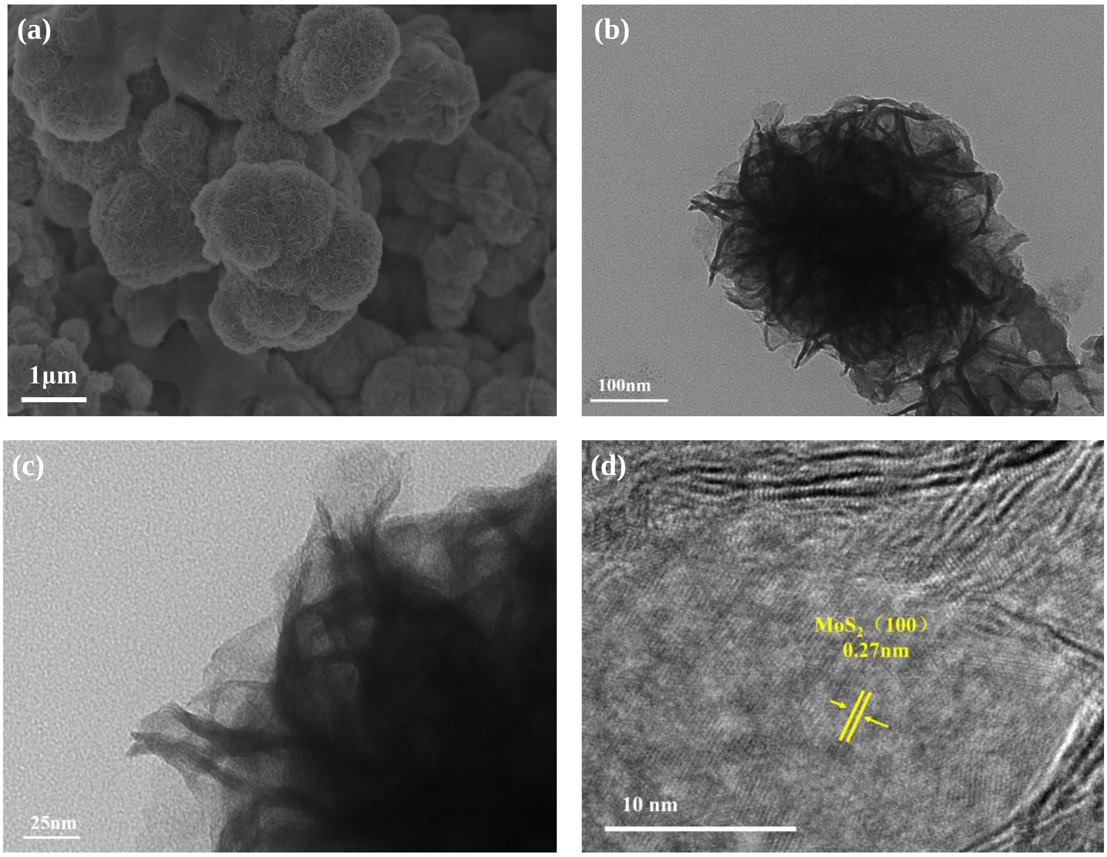

Fig. S2 (**a**) SEM image of the nanosheet-based MoS_2 inorganic nanoflower, (**b, c**) TEM images of MoS_2, (**d**) HRTEM image of MoS_2

The HRTEM showed fringes with a lattice spacing of 0.27nm, which corresponds to the (100) pane of MoS_2.

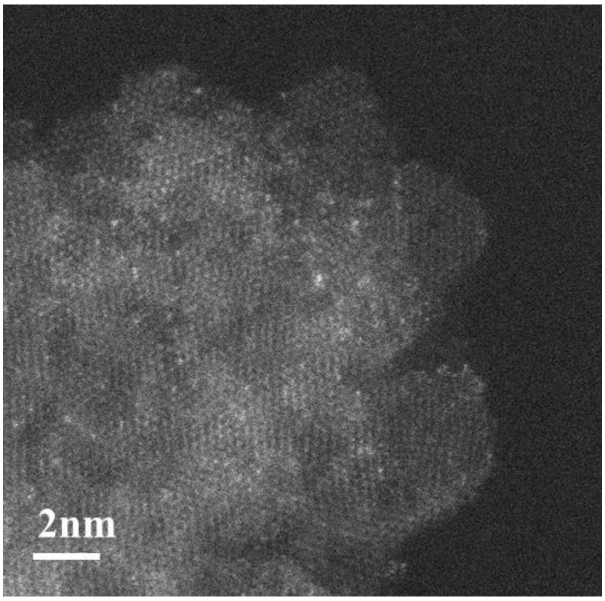

Fig. S3 HAADF-STEM image of Pt_1-MoS_2

The high-angle annular dark-field scanning transmission electron microscopy (HAADF-STEM) image shows the atomic dispersion of Pt species over the Pt_1-MoS_2 surface and no notable Pt clusters are observed.

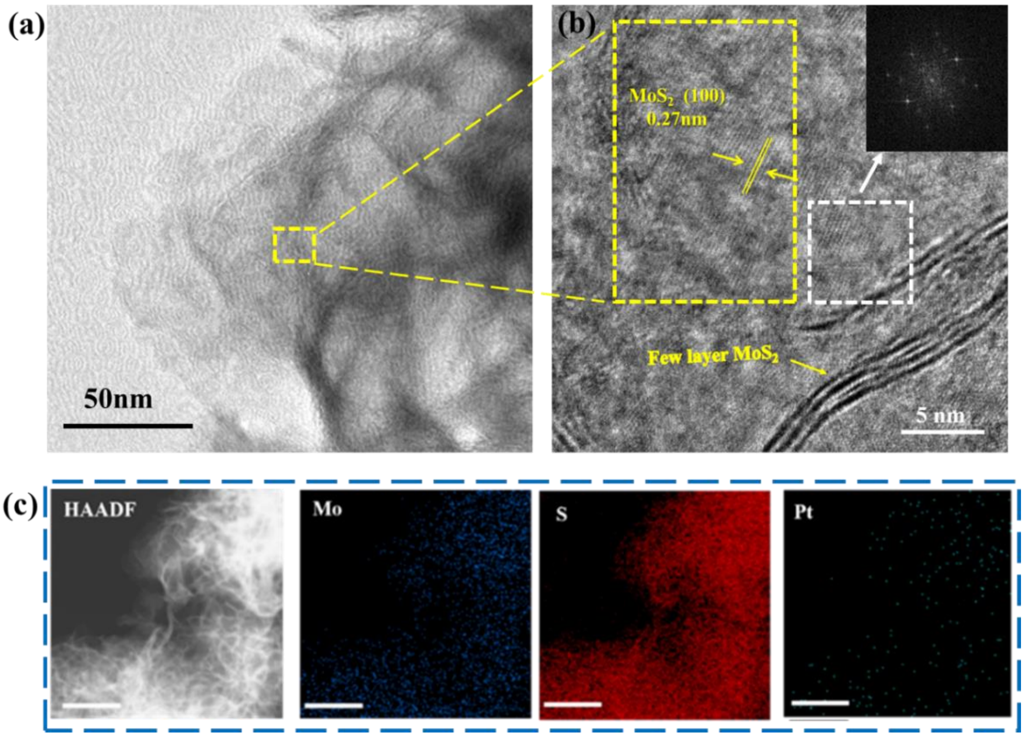

Fig. S4 (**a**) TEM image of the Pt_1-MoS_2-def, (**b**) HRTEM image of the Pt_1-MoS_2-def and the corresponding FFT diffraction pattern from the white frame in (**a**), (**c**) EDS elemental mapping of Pt_1-MoS_2-def

No Pt nanoparticles are found (Fig. S4a, b) in the Pt_1-MoS_2-def. Meanwhile, the energy-dispersive X-ray spectroscopy (EDS) mapping (Fig. S4c) further demonstrates the homogeneous distribution of Pt, Mo, and S elements.

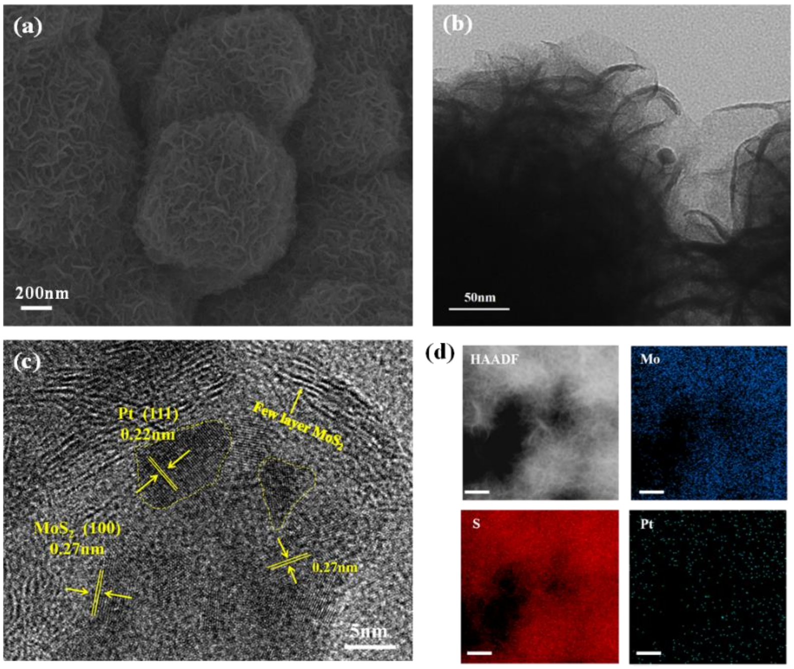

Fig. S5 (**a**) SEM image (**b**) TEM image (**c**) HRTEM (**d**) EDS elemental mapping of the Pt NPs-MoS_2

The HRTEM showed fringes with a lattice spacing of 0.22nm, which corresponds to the (111) pane of Pt nanoparticles and the lattice fringes with the interplanar distance of 0.27 nm are attributed to the (100) plane of MoS_2. The results showed that Pt nanoparticles are successfully modified on the surface of MoS_2.

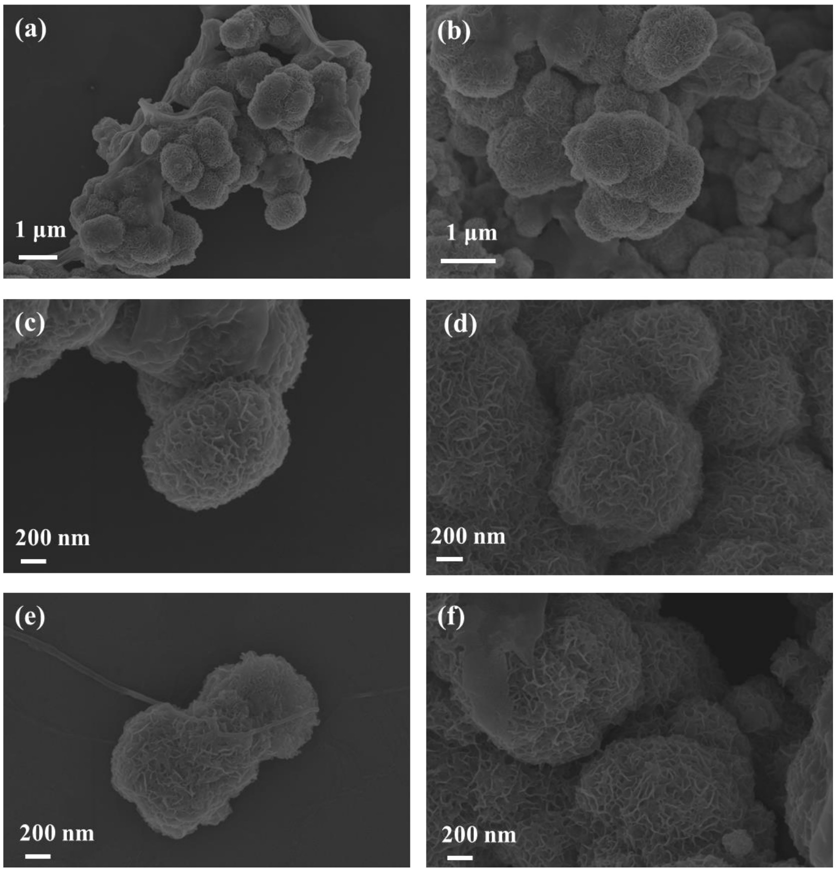

Fig. S6 SEM image of (**a, b**) Pt_1-MoS_2, (**c, d**) Pt_1-MoS_2-def, (**e, f**) Pt NPs-MoS_2

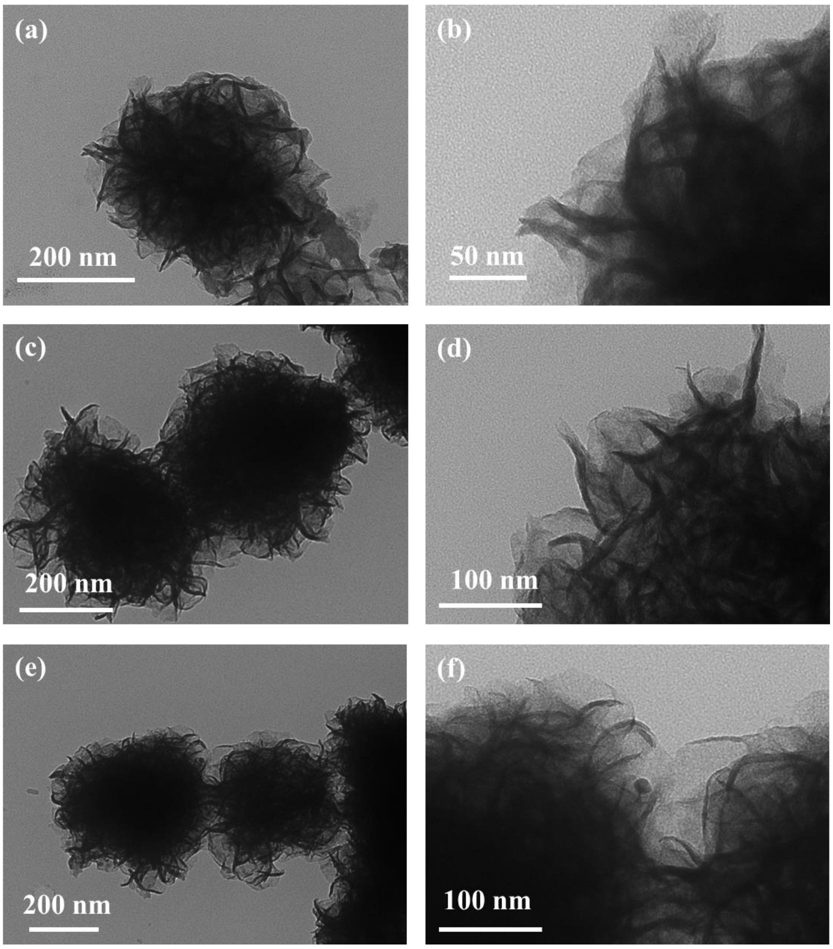

Fig. S7 TEM image of (**a, b**) Pt_1-MoS_2, (**c, d**) Pt_1-MoS_2-def, (**e, f**) Pt NPs-MoS_2

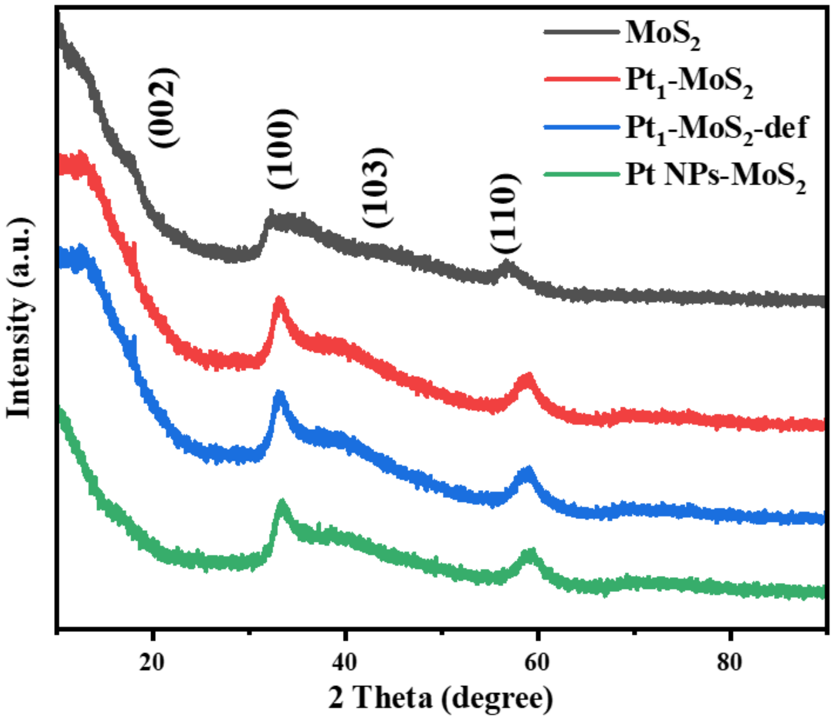

Fig. S8 XRD pattern of MoS_2, Pt_1-MoS_2, Pt_1-MoS_2-def and Pt NPs-MoS_2

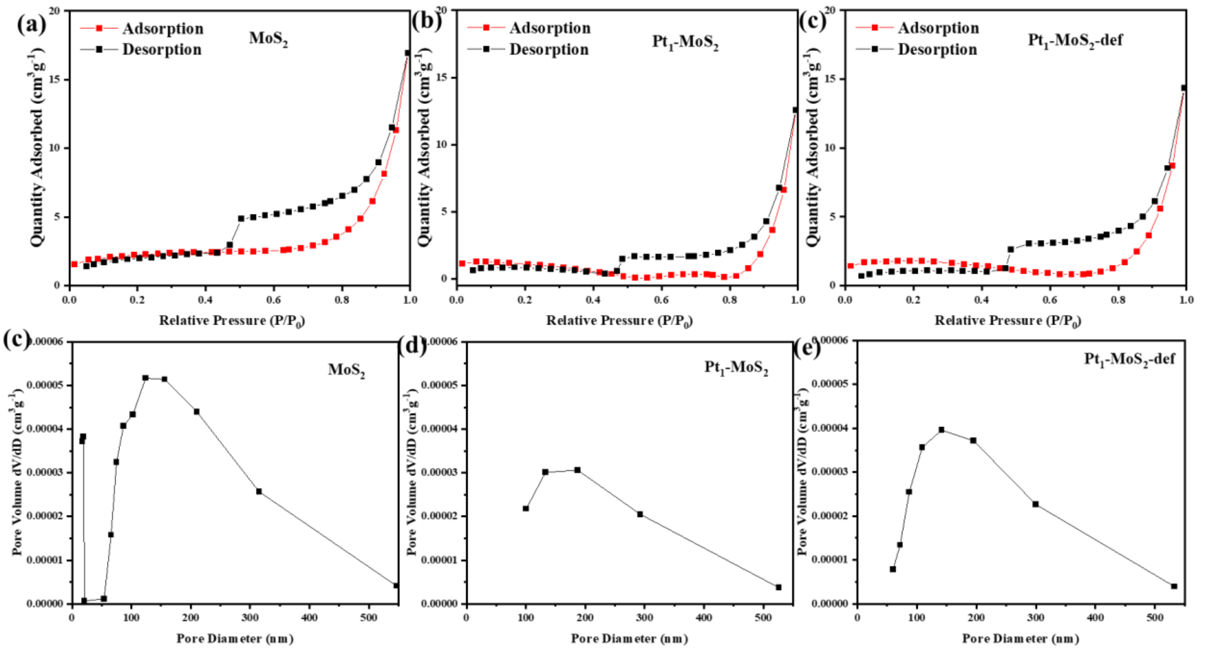

Fig. S9 (**a-c**) BET analysis showing a typical N2 adsorption-desorption isotherm of the MoS2, Pt1-MoS2 and Pt1-MoS2-def. (**d-f**) The pore size distribution curve of the MoS2, Pt1-MoS2 and Pt1-MoS2-def.

The Brunauer-Emmett-Teller (BET) surface area of MoS_2, Pt_1-MoS_2 and Pt_1-MoS_2-def measured by N_2 adsorption-desorption is 7.31 m^2 g^{-1}, 2.51 m^2 $g^{-1}$and 4.88 m^2 g^{-1}, respectively. The difference of BET surface area is insignificant. In addition, no obvious pores are found in three samples.

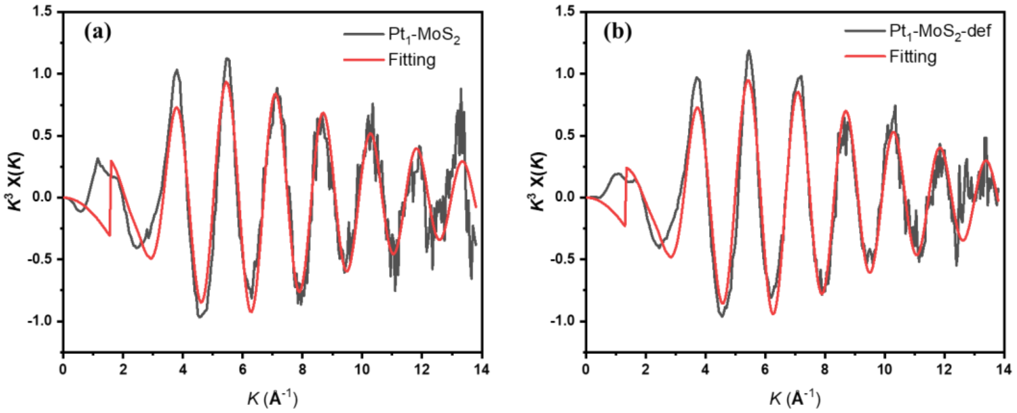

Fig. S10 Pt L_3-edge EXAFS k space fitting curve (red) and the experimental curve (black) of Pt_1-MoS_2 and Pt_1-MoS_2-def

DFT calculations

The calculation of "referenced" energies is done according to the following formulas:

Atomisation energy: $E_{atm} = E_{sys} - N_{Mo} \times E_{Mo\ atom} - N_S \times E_{S\ atom}$

Bond energy: $E_{bond} = E_{atomisation} / (2 \times N_{Mo} + N_S)$

Vacancy formation energy: $\triangle E_{vac} = E_{sys} - N_{Mo} \times (E_{Mo\ atom} + 2 \times E_{bond}) - N_S \times (E_{S\ atom} + E_{bond})$

Binding energy: $BE = E_{Slab+Ads} - E_{Slab} - E_{Ads}$

Species in Vacuum:

**Species**	**Energy (eV)**
**S atom**	-0.170
**Mo atom**	-0.388
**Pt atom**	-0.248

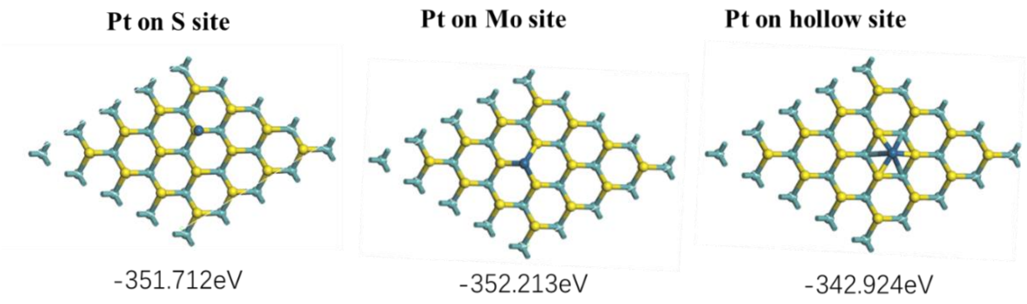

	Energy (eV)	Atomisation energy (eV)	Pt binding Energy (eV)
Pt on S site	**-351.712**	**-339.816**	**-2.764**
Pt on Mo site	**-352.213**	**-340.317**	**-3.265**
Pt in hollow site	**-342.924**	**-331.028**	**6.024**

Fig. S11 Geometrical structures of Pt_1-MoS_2 and their Pt binding energy

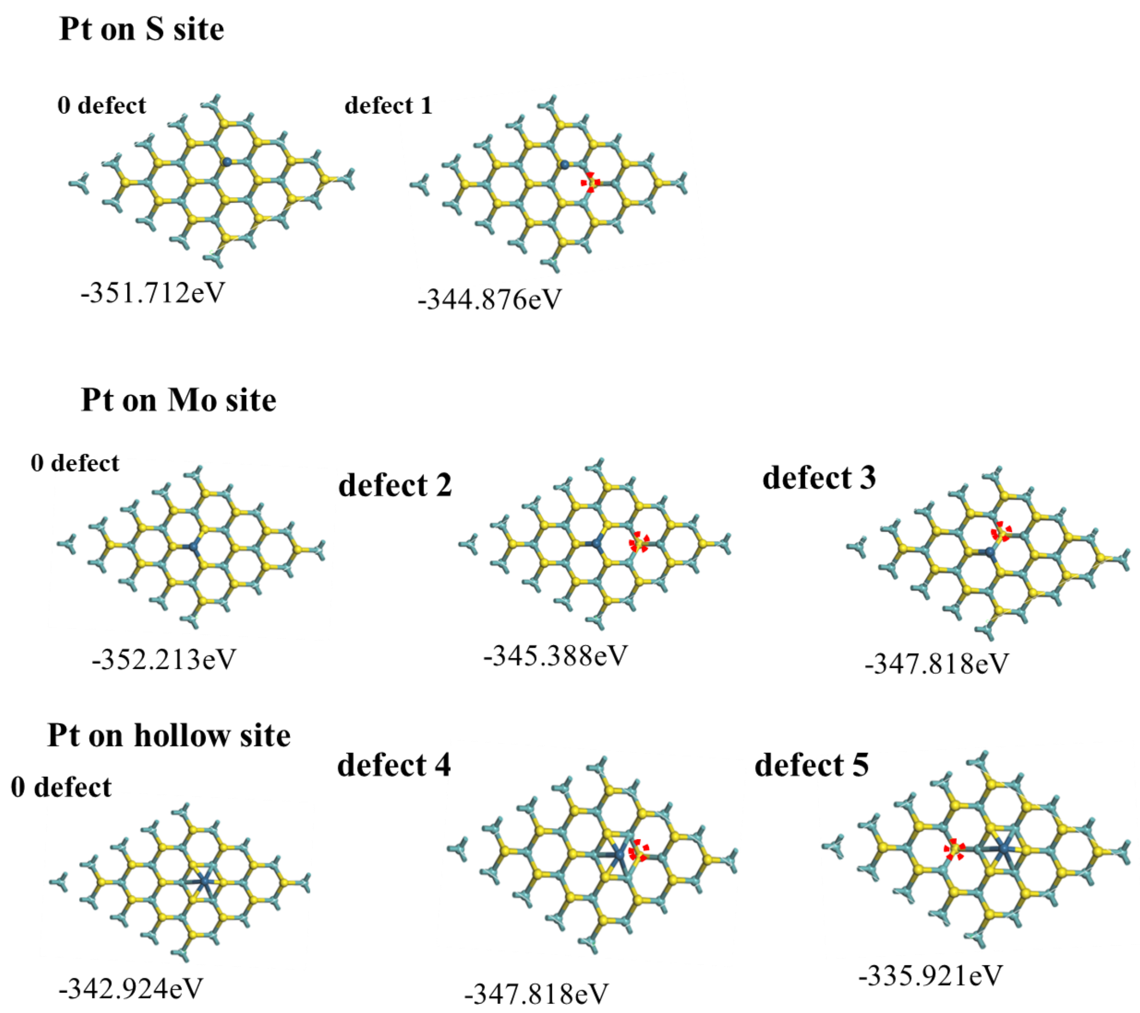

	Energy (eV)	Atomisation energy (eV)	Vacancy formation energy (eV)
**Defect 1**	**-344.876**	**-333.150**	**-1.364**
**Defect 2**	**-345.388**	**-333.662**	**-1.876**
**Defect 3**	**-347.818**	**-336.082**	**-4.306**
**Defect 4**	**-347.818**	**-336.092**	**-4.306**
**Defect 5**	**-335.921**	**-324.195**	**7.591**

Fig. S12 Geometrical structures of proposed Pt_1-MoS_2-def and S vacancy formation energy

According to the fitting results of EXAFS (Table S2), it can be observed that the coordination between Pt and S atoms predominates in the Pt_1-MoS_2-def sample, with a coordination number of three for the Pt-S bonds. Despite the fact that the defect 3 model has a lower vacancy formation energy (-4.306 eV), the defect 2 model exhibits better compatibility with the Pt coordination environment in the actual sample.

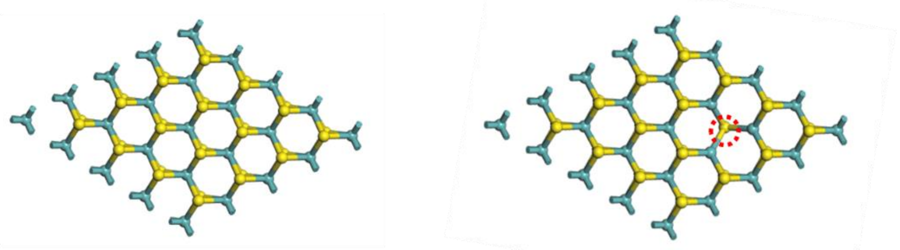

	Energy (eV)	Atomisation energy(eV)	Bond energy(eV)	Vacancy formation energy(eV)
MoS_2	**-348.700**	-337.052	-5.266	
MoS_2 with 1 S-vacancy	-341.934	-330.456		1.329

Fig. S13 Geometrical structures of MoS_2 and MoS_2 with 1 S-vacancy and its vacancy formation energy

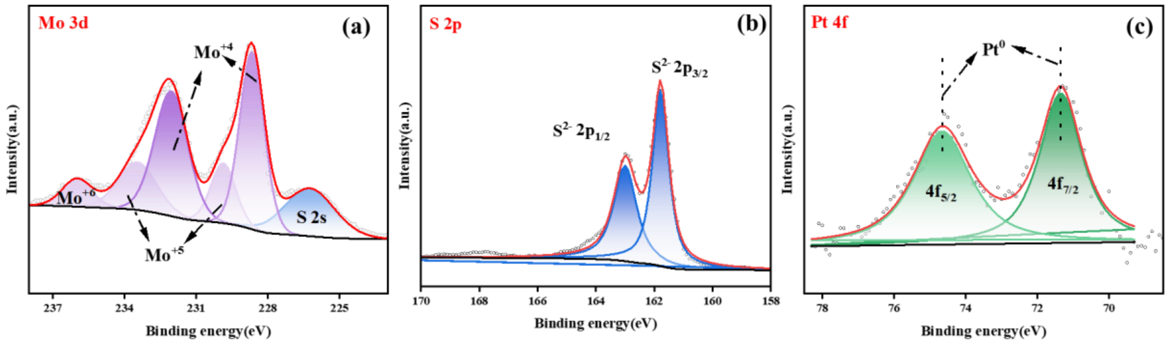

Fig. S14 (**a**)The Mo 3d (**b**) S 2p (**c**) Pt 4f XPS of Pt NPs-MoS_2

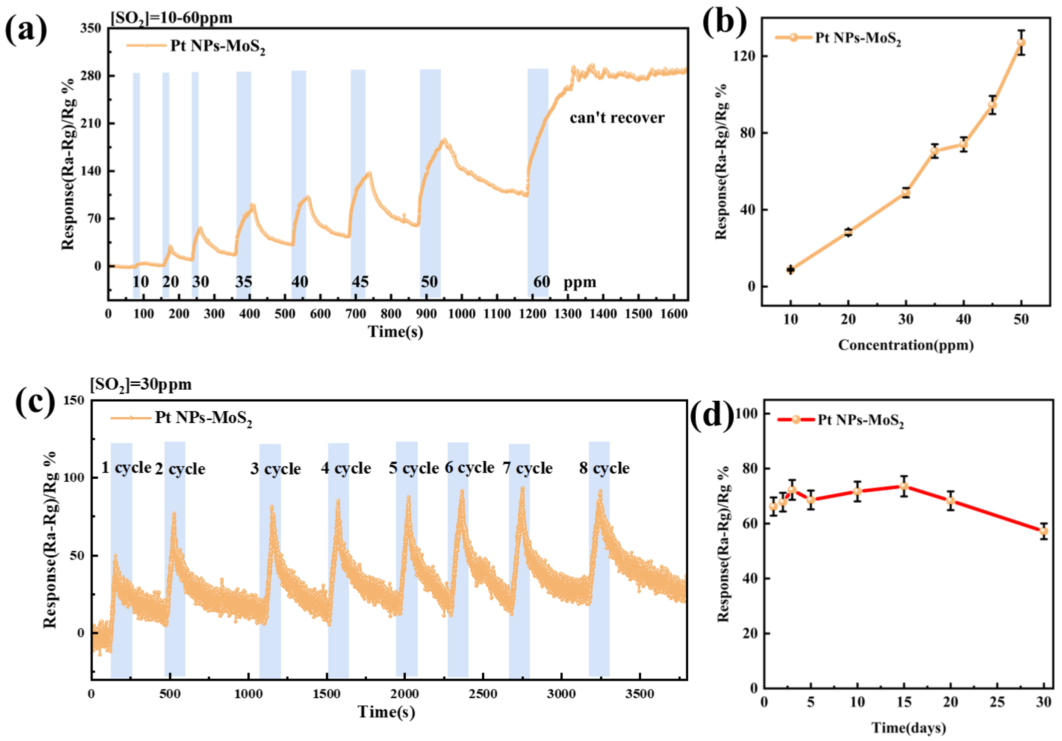

Fig. S15 (**a**) Dynamic response transitions and the (**b**) corresponding response of Pt NPs-MoS_2 sensors upon exposure to 10-60 ppm SO_2 gas, (**c**) Reliability tests using sensors upon 8 cyclic exposures to 30 ppm SO_2, (**d**) long-term stability toward 30 ppm SO_2 gas for Pt NPs-MoS_2 sensors

The dynamic response transition curves of the Pt NPs-MoS_2 sensors along with SO_2 concentrations in the range of 10-60 ppm were exhibited in Fig. S15(a). The inability of the Pt NPs-MoS_2 sample to recover at 60 ppm may be attributed to the saturation of active adsorption sites on the sample surface by SO_2. Fig. S15(b) shows the linear relationships between responses and SO_2 concentrations within 10-60 ppm for Pt NPs-MoS_2 sensors. Compared with Pt_1-MoS_2-def, the LOD of Pt NPs-MoS_2 is relatively high (10 ppm).

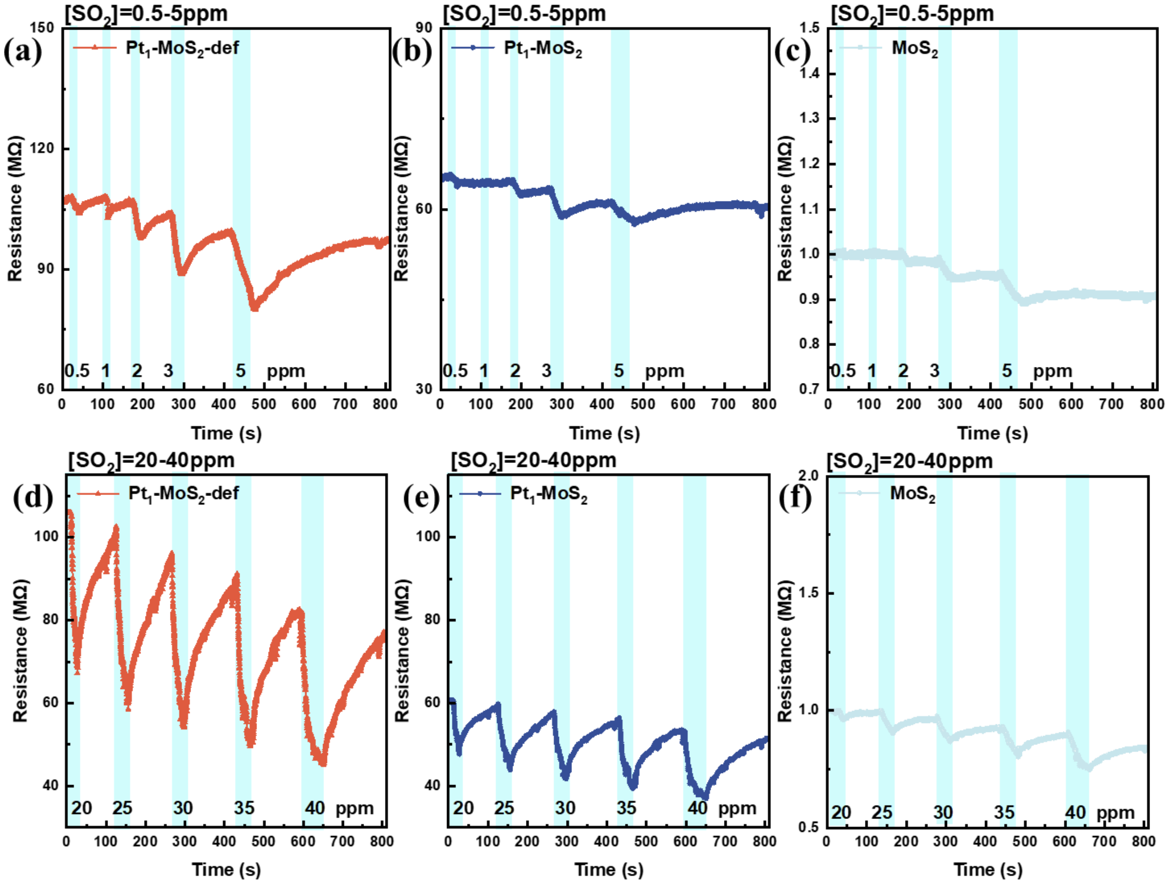

Fig. S16 The dynamic response-recovery resistance curves of MoS_2, Pt_1-MoS_2 and Pt_1-MoS_2-def sensors to different concentrations of SO_2 at RT

Obviously, the Pt_1-MoS_2-def sensor displays the highest response values toward different concentrations of SO_2 compared with Pt_1-MoS_2 and MoS_2 sensors.

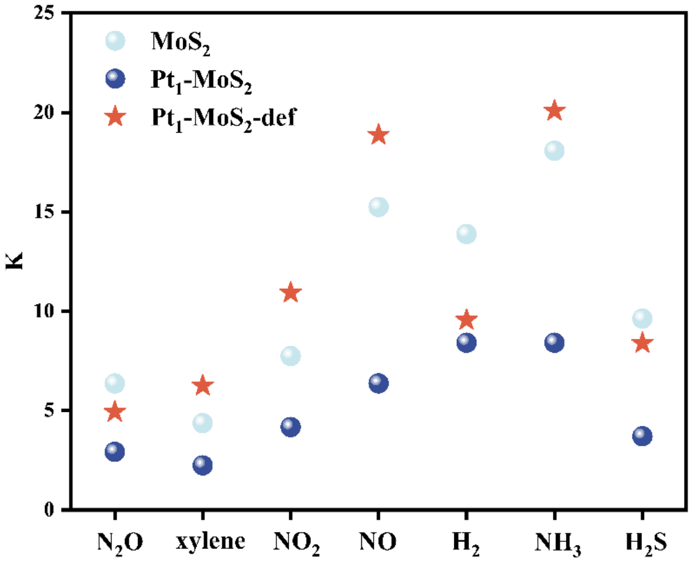

Fig. S17 K value of MoS_2, Pt_1-MoS_2 and Pt_1-MoS_2-def sensors to 3 ppm SO_2 at room temperature

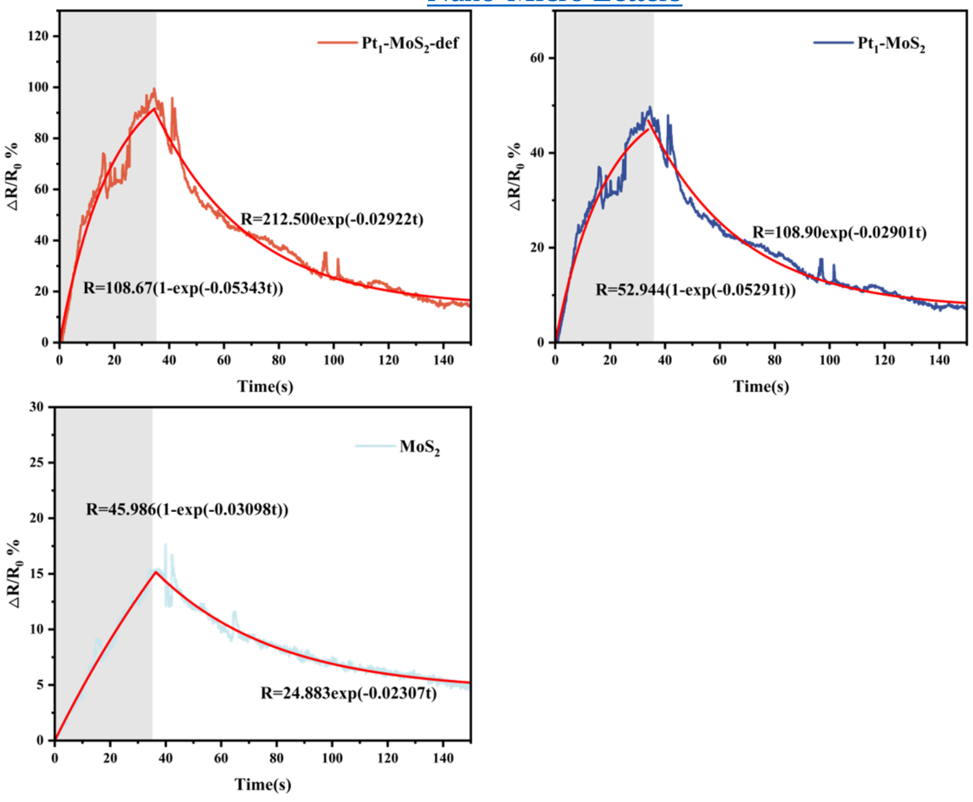

Sensor materials	K_{ads}($ppm^{-1}S^{-1}$)	K_{des}(S^{-1})	K (K_{ads}/K_{des}, ppm^{-1})
MoS_2	$3.164 \cdot 10^{-4}$	0.02307	0.0137
Pt_1-MoS_2	$9.563 \cdot 10^{-4}$	0.02901	0.0330
Pt_1-MoS_2-def	$9.684 \cdot 10^{-4}$	0.02922	0.0331

Fig. S18 Dynamic response transitions and the corresponding response/recovery fitting curves of the sensors for 25 ppm of SO_2 sensing

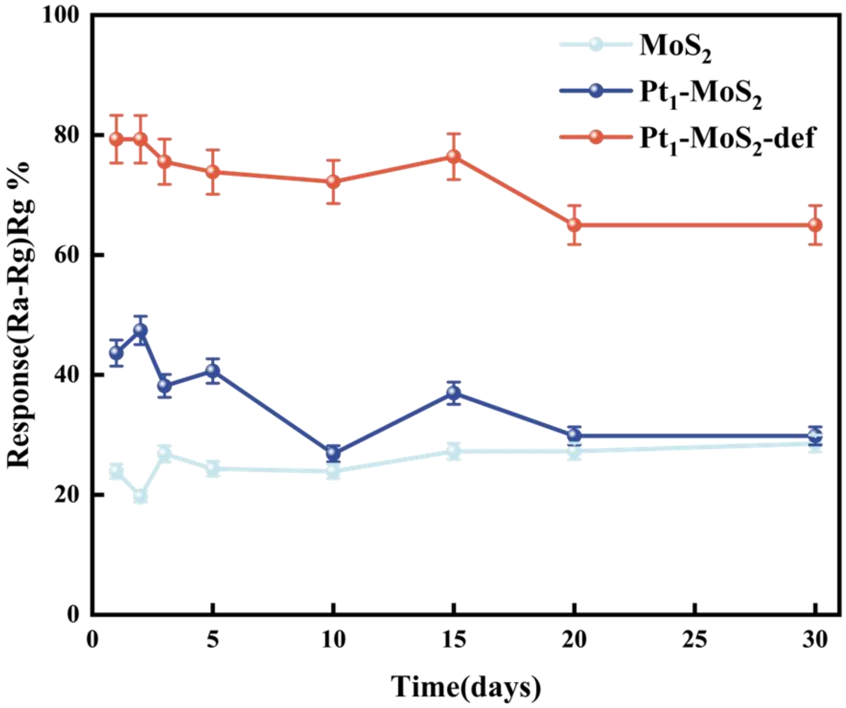

Fig. S19 Long-term stability toward 25 ppm SO_2 gas for MoS_2, Pt_1-MoS_2 and Pt_1-MoS_2-def sensors

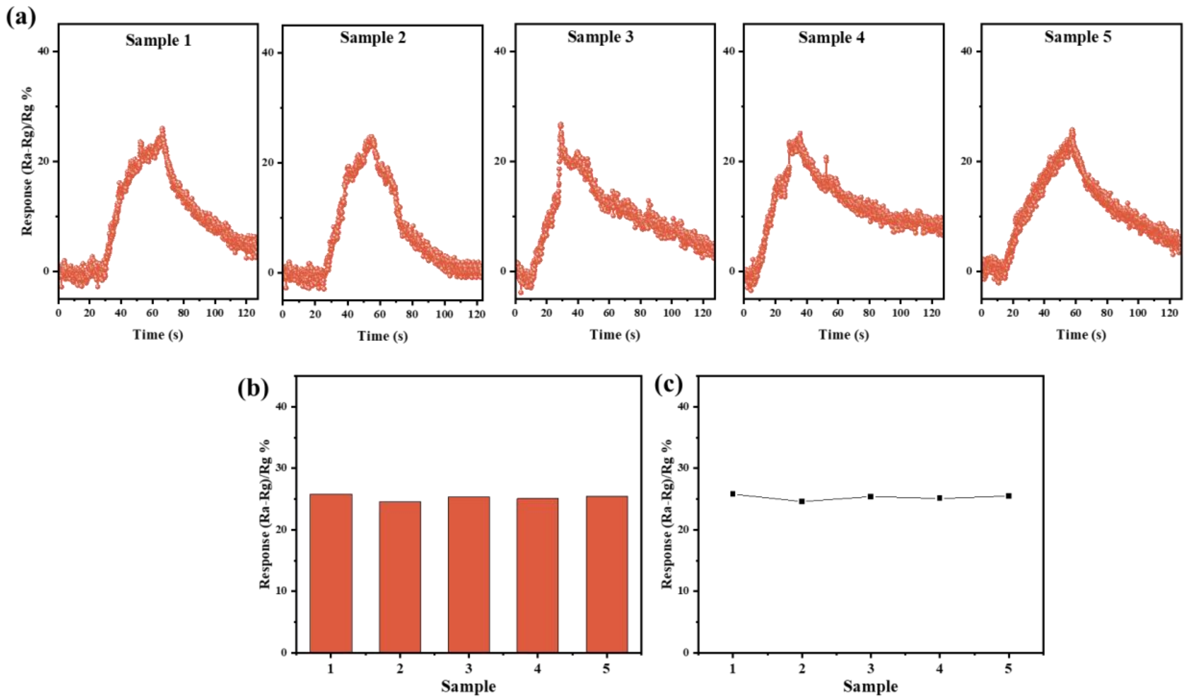

Fig. S20 (**a**) Time-related dynamic responses in the concentration 5 ppm SO_2. (**b, c**) Statistical graph of the response values for the five samples

The tests of Pt_1-MoS_2-def sensors in five sensors to 5 ppm of SO_2 are exhibited in (Fig. S20a). The response values of the five sensors to 5 ppm of SO_2 show uniformity, as depicted in Fig. S20 b, c, and the error among the five sensors is within 1%. Therefore, our experimental results and approach reflect statistics.

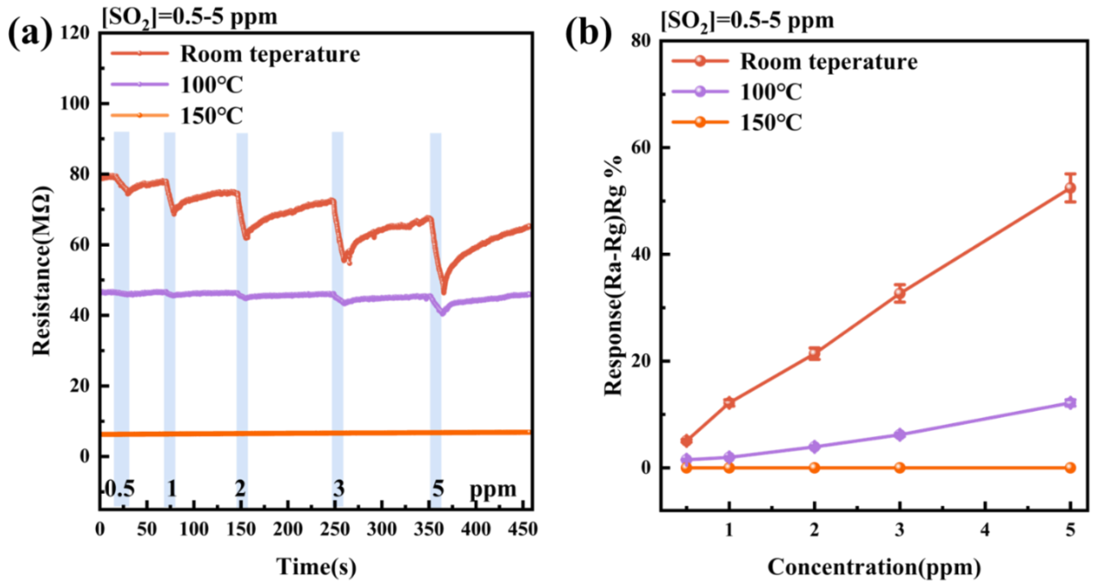

Fig. S21 (**a**) Dynamic resistance transitions and the (**b**) corresponding response of Pt_1-MoS_2-def sensors upon exposure to 0.5-5 ppm SO_2 gas under different temperature conditions, that is, RT, 50 and 100 °C

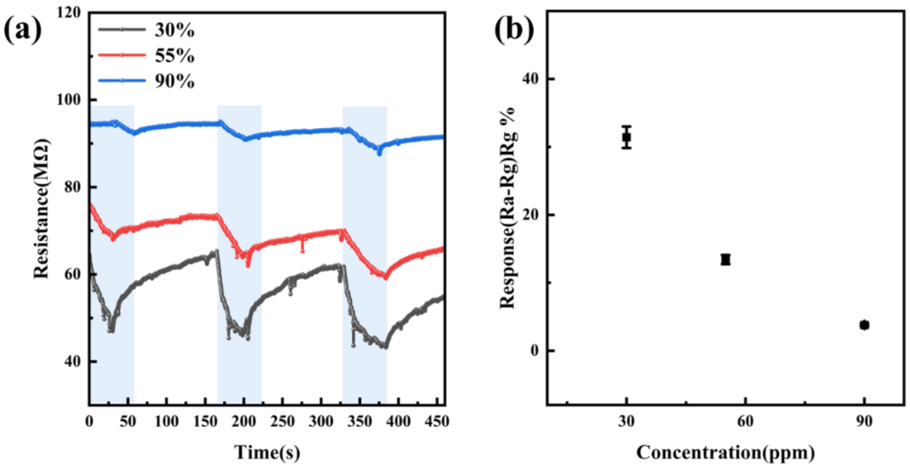

Fig. S22 (**a**) Dynamic resistance transitions and the (**b**) corresponding response of Pt_1-MoS_2-def sensors upon exposure to 5 ppm SO_2 gas under different humidity conditions, that is, 30%, 55% and 90% RH

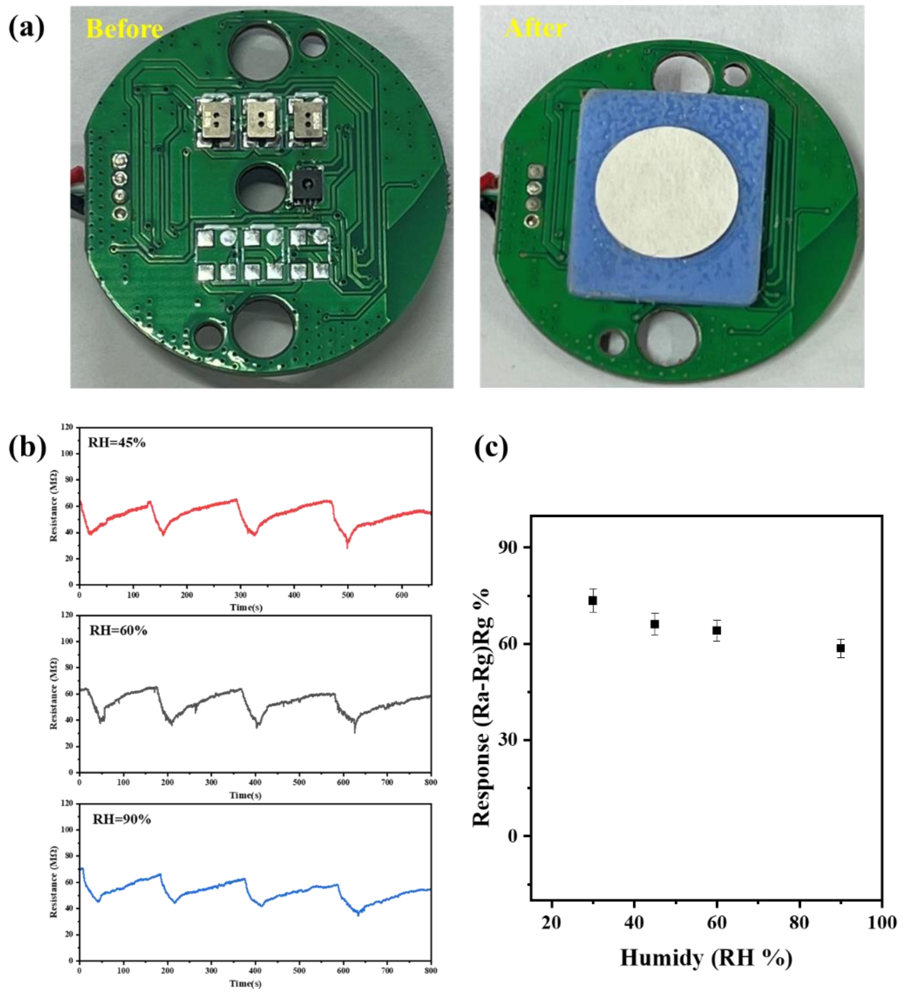

Fig. S23 (**a**)Schematic diagram of the improved device. The white circular disk represents the polytetrafluoroethylene film. (**b**) Dynamic resistance transitions and the (**c**) corresponding response of Pt_1-MoS_2-def sensors upon exposure to 25 ppm SO2 gas under different humidity conditions, that is 45%, 60% and 90% RH.

After adjusting the device, we tested the response of the material to 25 ppm SO_2 under different humidity conditions. It was observed that under 90% humidity, the sensor's response decreased by no more than 9%, effectively improving the normal operation of the device in high humidity environments.

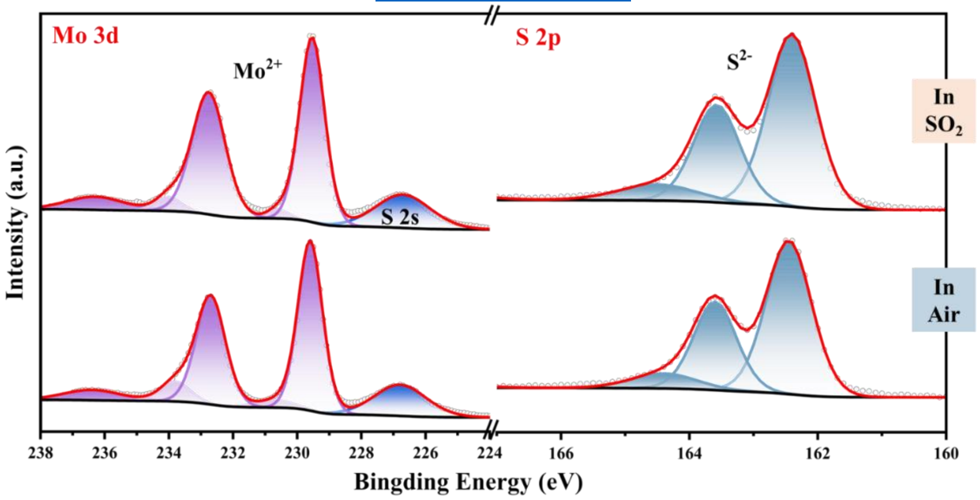

Fig. S24 *Ex situ* XPS spectra of MoS_2-def in the vicinity of Mo 3d and S 2p when exposed to air and SO_2 toward 100 ppm of SO_2 at room temperature, respectively

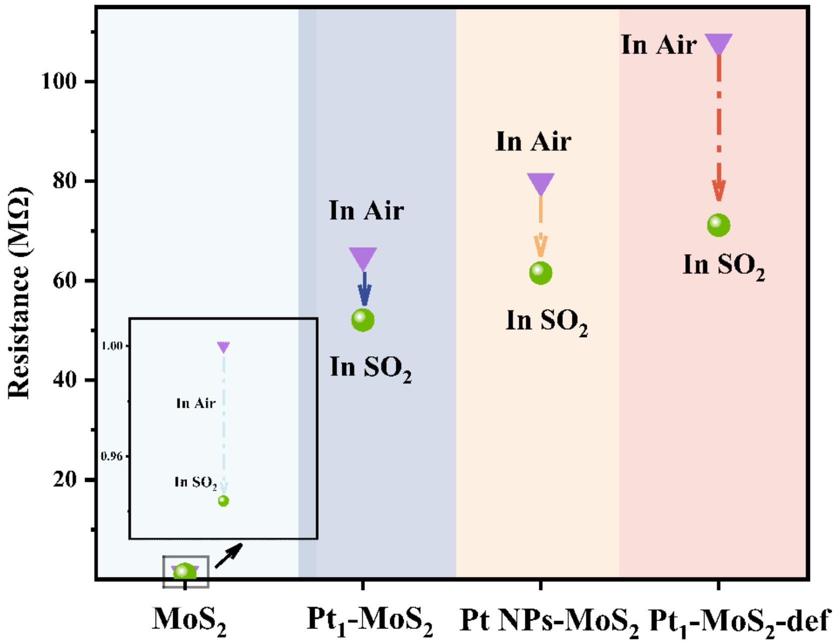

Fig. S25 Resistance changes of MoS_2, Pt_1-MoS_2, Pt NPs-MoS_2 and Pt_1-MoS_2-def during a gas sensitive response

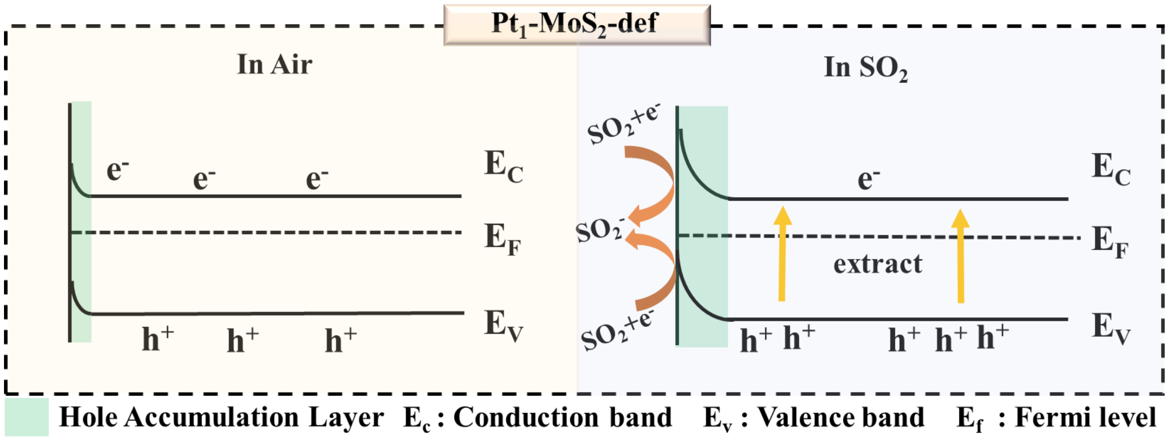

Fig. S26 Interface band structure of Pt_1-MoS_2-def

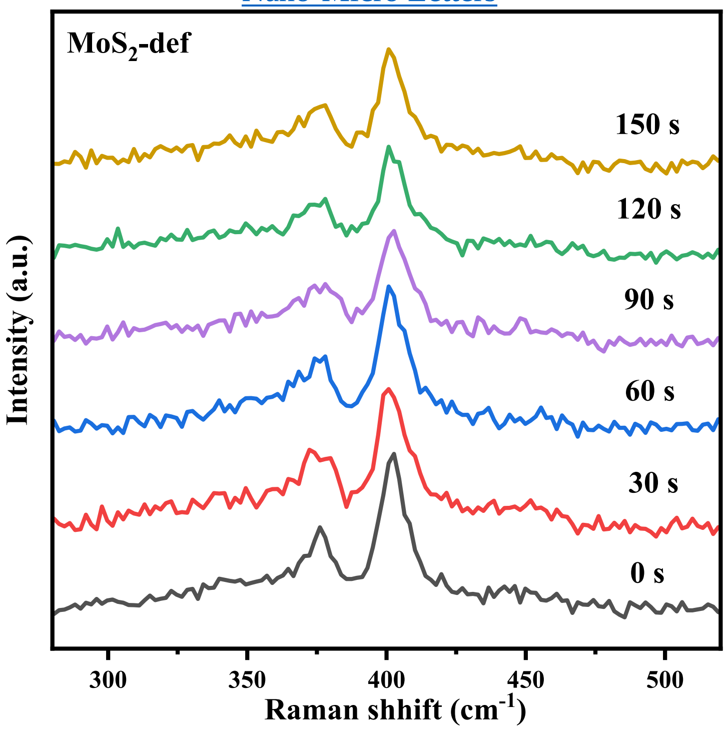

Fig. S27 *In situ* Raman spectra of the MoS_2-def

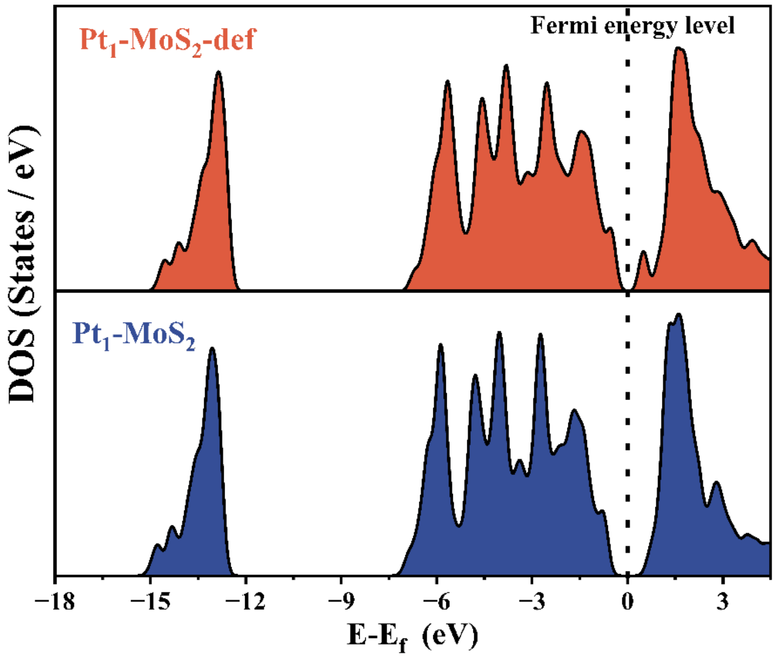

Fig. S28 The density of states (DOS) for Pt_1-MoS_2 and Pt_1-MoS_2-def

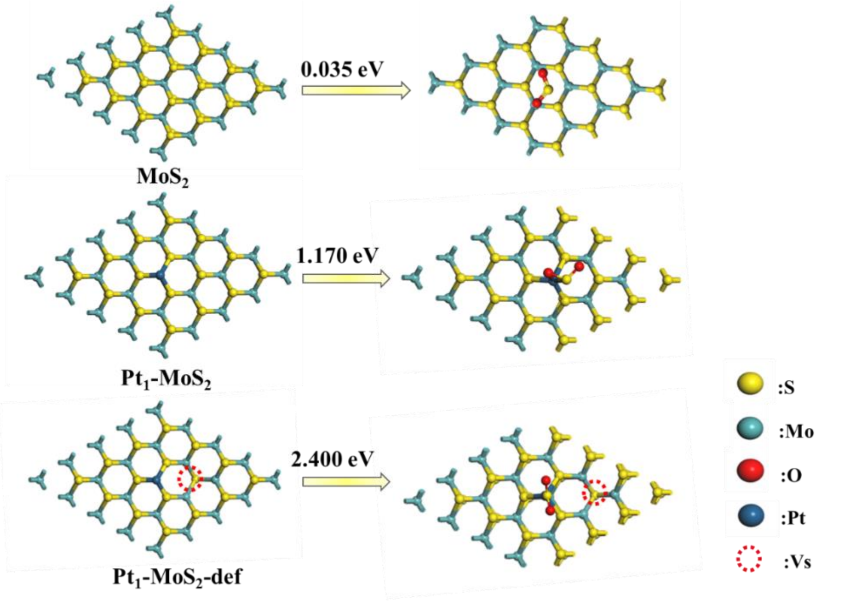

	E (eV)
MoS_2	-348.70029073
Pt_1-MoS_2	-352.16838034
Pt_1-MoS_2-def	-345.38811179
MoS_2+SO_2	-365.79717955
Pt_1-MoS_2+SO_2	-370.64013996
Pt_1-MoS_2-def+SO_2	-363.85677285
SO_2	-17.06178290

	E_{act}	E_{act}
MoS_2	0.035 eV	-3.5898kJ/mol
Pt_1-MoS_2	1.170 eV	-112.7314kJ/mol
Pt_1-MoS_2-def	2.400 eV	-135.0597kJ/mol

Fig. S29 Top views of the most stable adsorption configurations of SO_2 on MoS_2, Pt_1-MoS_2 and Pt_1-MoS_2-def

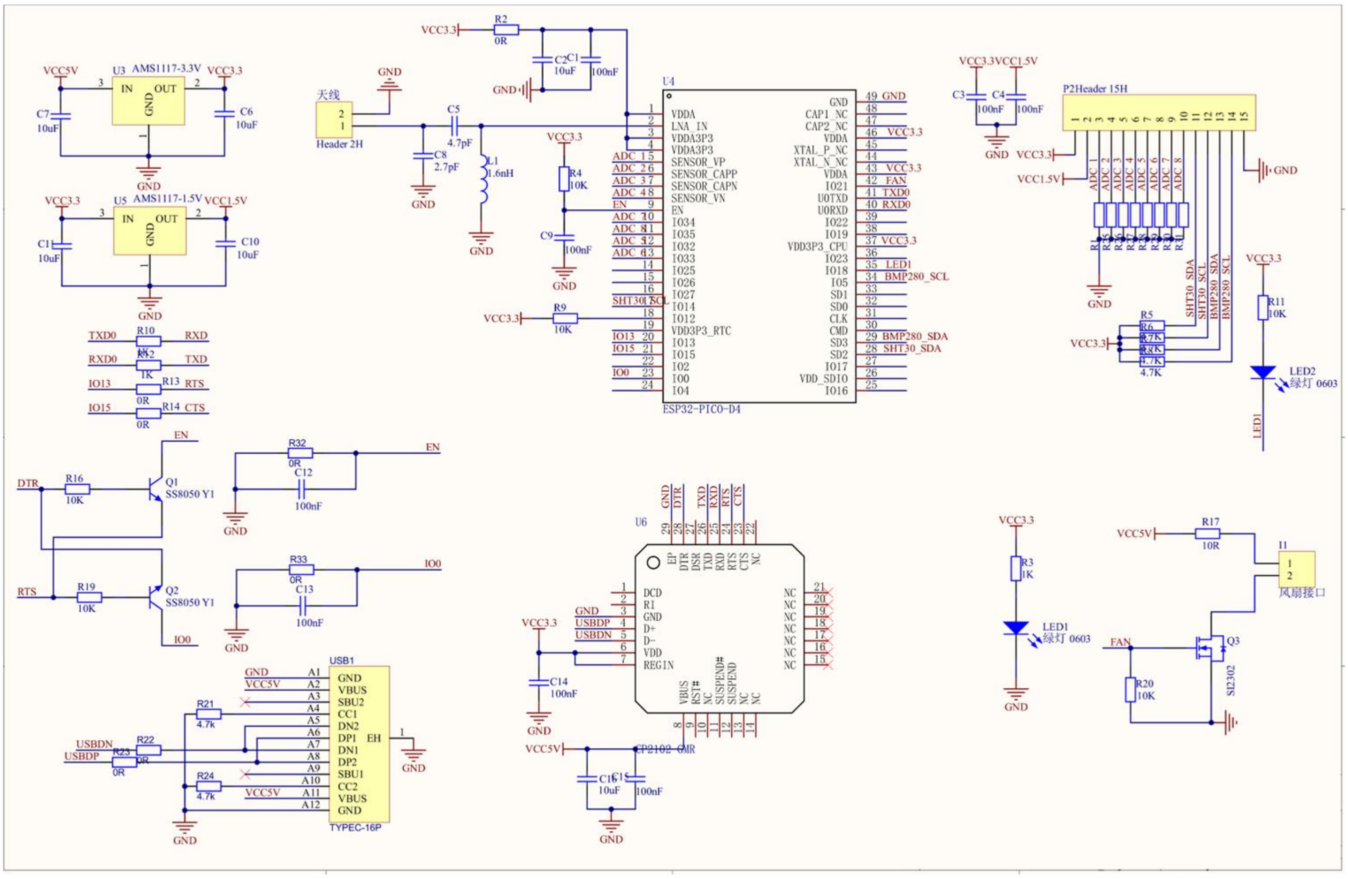

Fig. S30 The schematic circuit diagram of circuit board

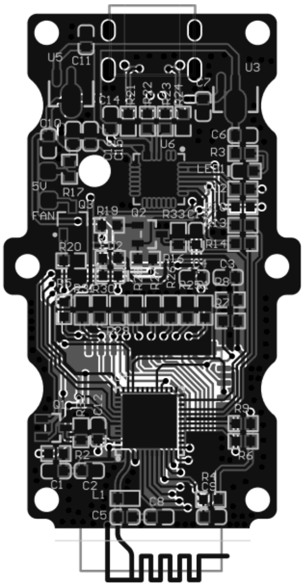

Fig. S31 The layouts of the printed circuit board (PCB)

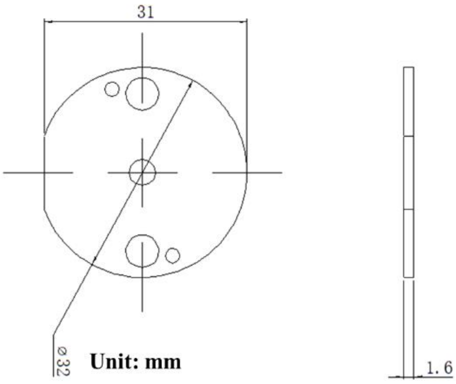

Fig. S32 Design survey drawing of disk-shaped device

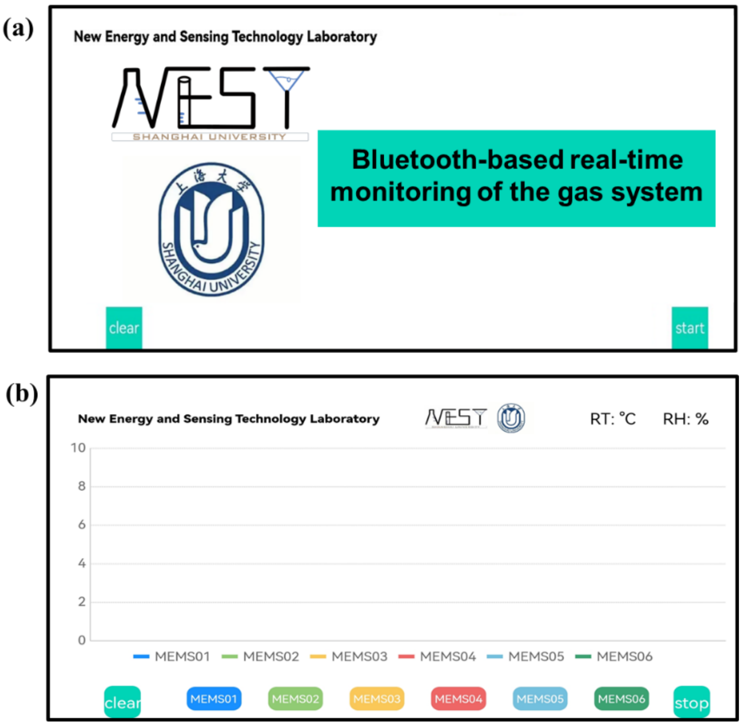

Fig. S33 Main interfaces of the custom mobile application. (**a**) The starting interface of the application. (**b**) The real-time data reading of SO_2 gas

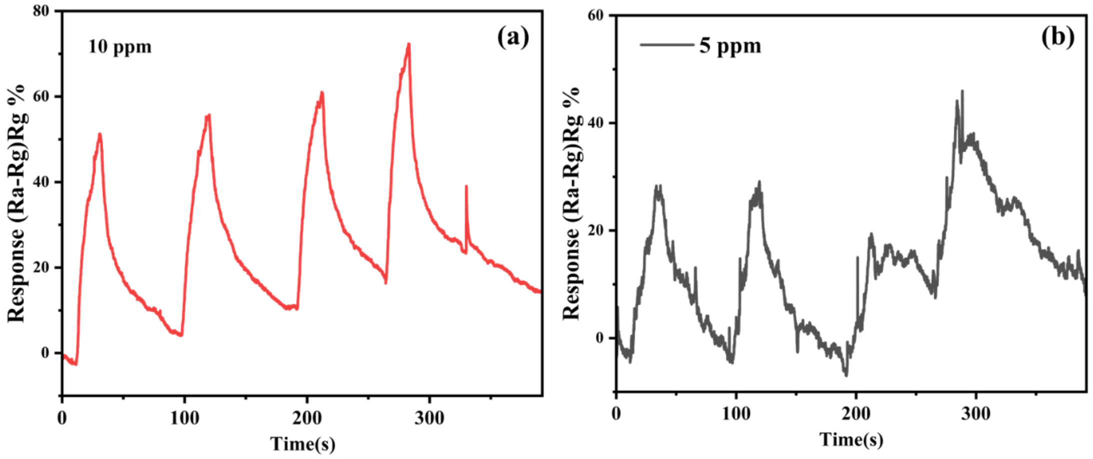

Fig. S34 Dynamic response transitions of Pt_1-MoS_2-def sensors upon exposure to 10 ppm and 5 ppm SO_2 gas

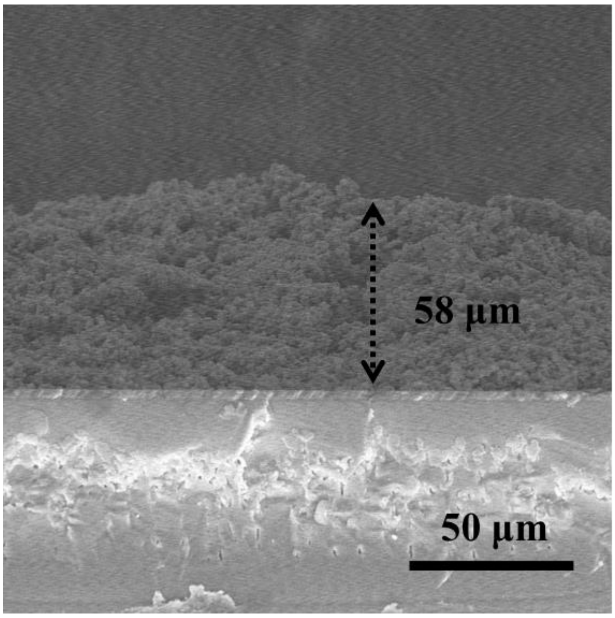

Fig. S35 SEM image of typical cross-section MEMS chips

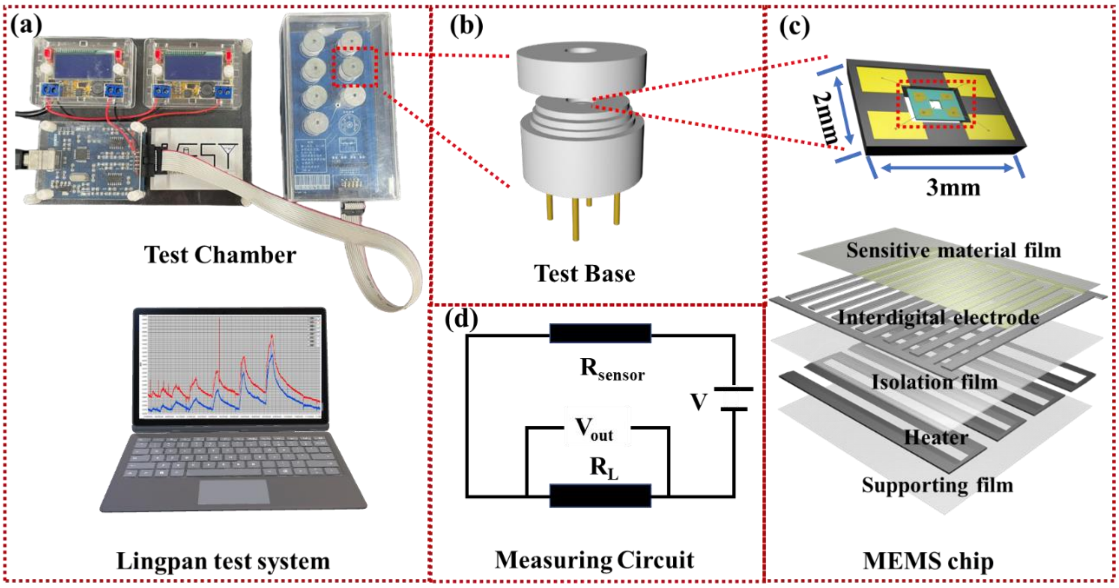

Fig. S36 Schematic of the fabrication for gas sensing system. (**a**) the gas sensing system LP-002A, (**b**) the exploded views of test base, (**c**) MEMS sensor, (**d**) measuring circuit of MEMS sensors

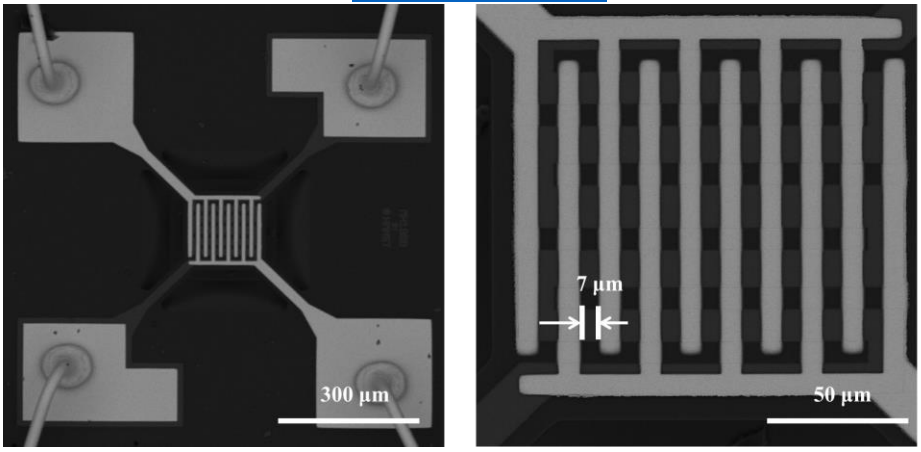

Fig. S37 SEM images of MEMS chips

The width and gap of the electrode is about 7 μm and the size of the interdigital electrode is 156 × 156 μm.

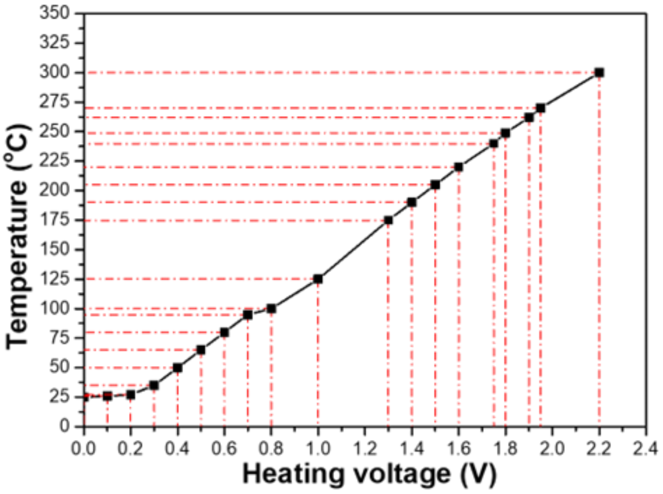

Fig. S38 The relations between temperature and heating voltage

Table S1 The content of noble metals in the obtained composites was measured by ICP-OES

**Samples**	**Pt (μg/ml)**	**Content of Pt (wt%)**
MoS_2	/	/
Pt NPs-MoS_2	0.0296	0.1
Pt_1-MoS_2-def	0.0299	0.1

Table S2 EXAFS fitting parameters at the Pt *L3*-edge for various samples （S_0^2=0.81）

**Sample**	**Coordination**	**CN[a]**	**R(Å)[b]**	**σ^2(Å^2)[c]**	**ΔE(eV)[d]**	**R-factor**
Pt foil	Pt-Pt	12	2.48	--	--	0.002
PtS_2	Pt-S	6	2.40	--	--	0.011
Pt_1-MoS_2-def	Pt-S	3.5	2.33+-0.01	0.0027+-0.0014	9.2	0.009
Pt_1-MoS_2	Pt-S	3.6	2.32+-0.01	0.0030+-0.0011	6.7	0.009

[a]*CN*, coordination number; [b]*R*, the distance to the neighboring atom; [c]σ^2, the Mean Square Relative Displacement (MSRD); [d]ΔE_0, inner potential correction; *R* factor indicates the goodness of the fit. $S0^2$ was fixed to 0.810, according to the experimental EXAFS fit of Ni foil by fixing *CN* as the known crystallographic value. * This value was fixed during EXAFS fitting, based on the known

structure of Pt. Fitting range: $2.9 \leq k$ (/Å) ≤ 12.6 and $1.0 \leq R$ (Å) ≤ 3.0 (Pt foil); $3.2 \leq k$ (/Å) ≤ 11.4 and $1.1 \leq R$ (Å) ≤ 2.4 (sample). A reasonable range of EXAFS fitting parameters: $0.700 < S_0^2 < 1.000$; $CN > 0$; $\sigma^2 > 0$ Å^2; $|\Delta E_0| < 10$ eV; R factor < 0.02.

Table S3 The of QCM tests

Sample	Fundamental frequency (10^6 hz)	Frequency after coating (10^6 hz)	Frequency shift (hz)	Load mass (ng)
**MoS_2**	10002396.76	10000337.85	2058.91	1143.838889
**Pt_1-MoS_2**	10002459.02	10000639.8	1819.22	1010.677778
**Pt_1-MoS_2-def**	10002432.37	10002178	254.37	141.3166667
**Pt NPs-MoS_2**	10002434.07	10001908.42	525.65	292.0277778

Table S4 A summary of SO_2 gas sensors at room temperate

Sensing material	Limit of detection	Operating temperature	Response （%）	References
**Ni-MoS_2**	250 ppb	RT	7.4 (5 ppm)	[S6]
**NiO-SnO_2**	5 ppm	RT	8.3 (50 ppm)	[S7]
**PANI**	10 ppm	RT	4.2 (10 ppm)	[S8]
**GO**	5 ppm	RT	6 (5 ppm)	[S9]
**ZnO**	100 ppm	RT	0.2 (100 ppm)	[S10]
**SnO_2-PANI**	2ppm	RT	3.1(4 ppm)	[S8]
**WO_3-PANI**	5ppm	RT	4.3(5 ppm)	[S11]
**RGO/WO_3**	50ppb	RT	30 (300ppb)	[S12]
**MWCNTs/WO_3**	50ppb	RT	23 (300ppb)	[S12]
**Zr-MOF**	1ppm	RT	0.3 (50ppm)	[S13]
**UIO-66-NH_2**	1ppm	RT	1.5 (50ppm)	[S14]
**ZC-LDH-iPOV-3**	100ppm	RT	71.71 (100ppm)	[S15]
**TiO_2/rGO**	1ppb	RT	11.14 (5ppm)	[S16]
**Polyaniline**	10ppm	RT	4.2 (10ppm)	[S10]
**Polyaniline-WO_3**	5ppm	RT	10.6 (10ppm)	[S17]
**Ag/PANI/SnO_2**	500 ppb	RT	2010 (50ppm)	[S18]
**ZnO/GaN**	500 ppb	RT	15 (10 ppm)	[S19]
**Pt_1-MoS_2-def**	500 ppb	RT	3.14 (500 ppb)	This work

S3 Supplementary References

[S1] X. Wang, W. Chen, L. Zhang, T. Yao, W. Liu et al., Uncoordinated amine groups of metal–organic frameworks to anchor single ru sites as chemoselective catalysts toward the hydrogenation of quinoline. J. Am. Chem. Soc. **139**, 9419 (2017). https://doi.org/10.1021/jacs.7b01686

[S2] G. Kresse and J. Furthmüller, Efficient iterative schemes for ab initio total-energy calculations using a plane-wave basis set. Phys. Rev. B **54**, 11169 (1996). https://doi.org/10.1103/PhysRevB.54.11169

[S3] J. P. Perdew, K. Burke, and M. Ernzerhof, Generalized gradient approximation made simple. Phys. Rev. Lett. **77**, 3865 (1996). https://doi.org/10.1103/PhysRevLett.77.3865

[S4] S. Grimme, J. Antony, S. Ehrlich, and H. Krieg, A consistent and accurate ab initio parametrization of density functional dispersion correction (DFT-D) for the 94 elements H-Pu. J. Chem. Phys. 132, 154104 (2010). https://doi.org/10.1063/1.3382344

[S5] S. Liu, Y. Yin, M. Wu, K. S. Hui, K. N. Hui et al., Phosphorus-mediated MoS_2 nanowires as a high-performance electrode material for quasi-solid-state sodium-ion intercalation supercapacitors. Small **15**, 1803984 (2019). https://doi.org/10.1002/smll.201900524

[S6] D. Zhang, J. Wu, P. Li, and Y. Cao, Room-temperature SO_2 gas-sensing properties based on a metal-doped MoS_2 nanoflower: an experimental and density functional theory investigation. J. Mater. Chem. A **5**, 20666 (2017). https://doi.org/10.1039/C7TA07001B

[S7] P. Tyagi, A. Sharma, M. Tomar, and V. Gupta, Metal oxide catalyst assisted SnO_2 thin film based SO_2 gas sensor. Sens. Actuators B Chem. **224**, 282 (2016). https://doi.org/10.1016/j.snb.2015.10.050

[S8] F. Shen, D. Wang, R. Liu, X. Pei, T. Zhang, and J. Jin, Edge-tailored graphene oxide nanosheet-based field effect transistors for fast and reversible electronic detection of sulfur dioxide. Nanoscale **5**, 537 (2012). https://doi.org/10.1039/C2NR32752J

[S9] R. Kumar, D. K. Avasthi, and A. Kaur, Fabrication of chemiresistive gas sensors based on multistep reduced graphene oxide for low parts per million monitoring of sulfur dioxide at room temperature. Sens. Actuators B Chem. **242**, 461 (2017).https://doi.org/10.1016/j.snb.2016.11.018

[S10] V. Chaudhary and A. Kaur, Solitary surfactant assisted morphology dependent chemiresistive polyaniline sensors for room temperature monitoring of low parts per million sulfur dioxide. Polym. Int. **64**, 1475 (2015). https://doi.org/10.1002/pi.4944

[S11] O. Lupan, L. Chow, and G. Chai, A single ZnO tetrapod-based sensor. Sens. Actuators B Chem. **141**, 511 (2009). https://doi.org/10.1016/j.snb.2009.07.011

[S12] P.-G. Su and Y.-L. Zheng, Room-temperature ppb-level SO_2 gas sensors based on RGO/WO_3 and MWCNTs/WO3 nanocomposites. Anal. Methods **13**, 782 (2021). https://doi.org/10.1039/D0AY02132F

[S13] X. Zhang, Z. Zhai, J. Wang, X. Hao, Y. Sun et al., Zr-MOF Combined with Nanofibers as an Efficient and Flexible Capacitive Sensor for Detecting SO_2. ChemNanoMat **7**, 1117 (2021). https://doi.org/10.1002/cnma.202100185

[S14] Z. Zhai, X. Zhang, J. Wang, H. Li, Y. Sun et al., Washable and flexible gas sensor based on UiO-66-NH_2 nanofibers membrane for highly detecting SO_2. Chem. Eng. J. **428**, 131720 (2022). https://doi.org/10.1016/j.cej.2021.131720

[S15] R.B. Shinde, N.S. Padalkar, S.V. Sadavar, A.S. Patil, S.B. Kale et al., Lattice engineering route for self-assembled nanohybrids of 2D layered double hydroxide with 0D isopolyoxovanadate: chemiresistive SO2 sensor. Mater. Today Chem. **24**, 100801 (2022). https://doi.org/10.1016/j.mtchem.2022.100801

[S16] D. Zhang, J. Liu, C. Jiang, P. Li, and Y. Sun, High-performance sulfur dioxide sensing properties of layer-by-layer self-assembled titania-modified graphene hybrid nanocomposite. Sens. Actuators B Chem. **245**, 560 (2017). https://doi.org/10.1016/j.snb.2017.01.200

[S17] V. Chaudhary and A. Kaur, Enhanced room temperature sulfur dioxide sensing behaviour of in situ polymerized polyaniline–tungsten oxide nanocomposite possessing honeycomb morphology. RSC Adv. **5**, 73535 (2015). https://doi.org/10.1039/C5RA08275G

[S18] H. Xu, J. Li, P. Li, J. Shi, X. Gao, and W. Luo, Highly efficient SO_2 sensing by light-assisted Ag/PANI/SnO_2 at room temperature and the sensing mechanism. ACS Appl. Mater. Interfaces **13**, 49194 (2021). https://doi.org/10.1021/acsami.1c14548

[S19] M.A.H. Khan, B. Thomson, J. Yu, R. Debnath, A. Motayed et al., Scalable metal oxide functionalized GaN nanowire for precise SO_2 detection. Sens. Actuators B Chem. **318**, 128223 (2020). https://doi.org/10.1016/j.snb.2020.128223

[S20] X. He, Z. Ying, F. Wen, L. Li, X. Zheng et al., MoS_2-doped spherical SnO_2 for SO_2 sensing under UV light at room temperature. Mater. Sci. Semicond. Process. **134**, 105997 (2021). https://doi.org/10.1016/j.mssp.2021.105997
